# Prednisolone add-on in early phase schizophrenia: A randomized, double-blind, placebo-controlled pilot study

**DOI:** 10.1016/j.bbih.2025.101047

**Published:** 2025-07-10

**Authors:** Gunnhild E. Hoprekstad, Erik Johnsen, Christoffer Bartz-Johannessen, Jill Kristin Bjarke, Petros Drosos, Ole Bernt Fasmer, Kristian Varden Gjerde, Solveig K. Reitan, Silje Skrede, Iris E.C. Sommer, Lena Stabell, Vidar M. Steen, Rune A. Kroken

**Affiliations:** a Division of Psychiatry, Haukeland University Hospital, Bergen, Norway; b Department of Clinical Medicine, University of Bergen, Bergen, Norway; c Mohn Research Center for Psychotic Disorders, Bergen, Norway; d Section of Clinical Pharmacology, Department of Medical Biochemistry and Pharmacology, Haukeland University Hospital, Bergen, Norway; e Department of Global Public Health and Primary Care, University of Bergen, Bergen, Norway; f St. Olav's University Hospital, Department of Mental Health, Nidelv DPS, Trondheim, Norway; g Norwegian University of Science and Technology, Department of Mental Health, Trondheim, Norway; h Dr. Einar Martens Research Group for Biological Psychiatry, Department of Clinical Science, University of Bergen, Bergen, Norway; i Department of Medical Genetics, Haukeland University Hospital, Bergen, Norway; j NKS Olaviken Gerontopsychiatric Hospital, Erdal, Norway; k University Medical Center Groningen, Groningen, the Netherlands; l TIPS-Network for Clinical Research in Psychosis, Clinic for Adult Mental Health, Stavanger University Hospital, Stavanger, Norway

**Keywords:** Schizophrenia, Psychosis, Inflammation, treatment, PANSS total, PANSS general, Prednisolone, RCT

## Abstract

**Background:**

New pharmacological treatment strategies are urgently needed for schizophrenia since current antipsychotic medications have limitations related to efficacy, tolerability, and disease course modification. Different lines of evidence converge on aberrant activity of the immune system, but translation into new treatment is still pending. Studies investigating the antipsychotic properties of less potent anti-inflammatory drugs have provided equivocal findings. A potent agent like prednisolone might be better suited for establishing proof-of-concept that dampening of inflammation may be beneficial in schizophrenia.

**Methods:**

In this double-blind multicentre pilot study, 12 patients (aged 18–39) with schizophrenia maintained on stable antipsychotic regimens were randomized to prednisolone or placebo. Study medication was initiated with 40 mg/day and gradually tapered to zero over a period of 6 weeks. The primary outcome was difference in the Positive and Negative Syndrome Scale (PANSS) total score 6 weeks after baseline.

**Outcomes:**

In patients randomized to prednisolone (N = 6), a clear trend towards more pronounced reduction of psychotic symptoms was observed compared to the placebo group (N = 6). Difference in symptom severity per the PANSS total score after 6 weeks of add-on treatment was 15·4 (SD = 8·5, p = 0·101). For the PANSS general score, we observed a statistically significant difference of 12·5 (SD = 4·6, p = 0·021) at week 6. Safety and tolerability were acceptable for the duration of prednisolone treatment.

**Interpretation:**

These findings suggest beneficial effect of add-on prednisolone. However, this needs further replication in a larger sample for confirmation and to identify the mechanisms of action.

## Abbreviations

AP –: antipsychotic medication
BBB –: blood brain barrier
BMI –: body mass index
CDSS –: Calgary Depression Scale for Schizophrenia
CGI-SS –: Clinical Global Impression for Severity of Suicidality
CRP –: C-reactive protein
DDD –: defined daily dose
DSM-IV –: Diagnostic and Statistical Manual of Mental Disorders, Fourth Edition
DUP –: Duration of untreated psychosis
FEP –: first episode of psychosis
GAF –: Global Assessment of Functioning scale, split version
GWAS –: genome-wide association study
HbA1c –: haemoglobin A1c
ICD-10 –: International Classification of Diseases, 10th Revision
ICH-GCP –: International Conference on Harmonization (ICH) of Good Clinical Practice (GCP)
M.I.N.I. –: Mini International Neuropsychiatric Interview
NorPEPS –: the Norwegian Prednisolone in Early Psychosis Study
PANSS –: Positive and Negative Syndrome Scale
RCT –: randomized controlled trial
SAE –: serious adverse event
SD –: standard deviation
YMRS –: Young Mania Rating Scale
WHO –: World Health Organization

## 1 Introduction

Schizophrenia, arguably the most severe mental disorder, has a lifetime risk of 1 % and typically develops in adolescence or early adulthood (Jauhar et al., 2022). A remitting–relapsing course is seen in most patients, and there is no indication of improved recovery rates through the decades (Jaaskelainen et al., 2013). At 10-year follow-up of a first-episode psychosis cohort, only 29 % had reached clinical recovery (Wold et al., 2024). Current antipsychotic drug treatment comprises partial or complete antagonism at striatal postsynaptic type 2 dopamine receptors, facilitating symptom reduction with a moderate to large effect size, but with no established disease-modifying effect (Howes et al., 2012; Ghaemi, 2022). Significant subgroups do not benefit from antipsychotics even at first-episode treatment, with increasing numbers of non-responders during subsequent relapses (Emsley et al., 2013). Moreover, cognitive dysfunction and negative symptoms such as apathy and social withdrawal are generally rather unresponsive to antipsychotic drugs (Jauhar et al., 2022; Husa et al., 2017). This is a major challenge since these clinical entities are significantly associated with long-term functional outcome and contribute substantially to the high disability rates in schizophrenia (Milev et al., 2005). Taken together, developing new treatment strategies with novel and broader therapeutic action that span beyond the dopaminergic system are strongly needed.

Components of the immune system have become putative treatment targets, as the presence of inflammation and immunological alterations has been robustly documented in schizophrenia (Kroken et al., 2018; Trubetskoy et al., 2022; Schizophrenia Working Group of the Psychiatric Genomics C, 2014; Horvath and Mirnics, 2014; Upthegrove et al., 2014; Gangadin et al., 2024). However, at this stage it is unclear what causes such disturbances, a fact which may underlie the modest success of intervention trials. Several clinical studies have investigated the efficacy and tolerability of different anti-inflammatory agents (Kroken et al., 2018). However, despite some promising findings, anti-inflammatory drugs in general have yet to prove therapeutic efficacy (Cakici et al., 2019; Fond et al., 2023). The cause of the missing translation from findings of a low-grade inflammatory state to the implementation of anti-inflammatory drug therapy in schizophrenia may be that most researched drugs have only modest anti-inflammatory potency and a rather narrow mechanism of action, often targeting the production of prostaglandins.

Corticosteroids are potent anti-inflammatory agents in almost all systems of the human body as part of treatment for both acute and chronic inflammation. One of them, prednisolone, is known for its potent and broad anti-inflammatory properties, affecting both the innate and adaptive compartments of the immune system and crossing the blood brain barrier (BBB) (Spies et al., 2010). Accordingly, prednisolone is a clinically relevant drug when the immunomodulatory approach to schizophrenia psychopathology is examined. There are some early reports of corticosteroids administered mono-therapeutically on the indication of schizophrenia, but few in the framework of randomized controlled trials (RCTs) (Polatin et al., 1955). Symptom improvement was not found in a recent RCT when adjunctive treatment with prednisolone was compared to a placebo, with a similar protocol to the present study (Nasib et al., 2020), but safety and tolerability was good (Nasib et al., 2021). The primary aim of the present study was to put the inflammation hypothesis of schizophrenia to the clinical test by using the potent anti-inflammatory agent prednisolone as an add-on treatment during antipsychotic drug therapy.

## 2 Methods

### 2.1 Study design

The Norwegian Prednisolone in Early Psychosis Study (NorPEPS) is a randomized, double-blind, placebo-controlled add-on study of prednisolone versus placebo. NorPEPS was performed at university hospitals in Bergen, Stavanger, and Trondheim. The study was approved by the Regional Committee for Medical Research Ethics Western Norway (2017/620) and by the Norwegian Medicines Agency (https://clinicaltrials.gov/study/NCT03340909?term=NorPEPS&rank=1).

### 2.2 Participants

After providing written informed consent, eligible patients aged 18–70 years, with a diagnosis within the schizophrenia spectrum (DSM-IV: 295∗) or psychosis not otherwise specified (298·9), were included, and medical doctors confirmed the diagnoses with the Mini International Neuropsychiatric Interview Plus (M.I.N.I.-Plus) (Sheehan et al., 1998). The first psychotic symptom onset could not be more than 7 years prior to study inclusion. The total score in the Positive and Negative Syndrome Scale (PANSS) (Kay et al., 1987) had to be 60 or above, indicating an active phase of psychosis. All study participants were prescribed a stable dose of antipsychotic medication for at least 3 weeks prior to the administration of study medication. Exclusion criteria included contraindications for the use of prednisolone according to the summary of product characteristics: heart failure, diabetes mellitus, osteoporosis, systemic infections, use of systemic glucocorticoids, carbamazepine, rifampicine, primidone, barbiturates, phenytoin, highly active antiretroviral therapy medication, telaprevir, or boceprevir. Pregnant or breastfeeding women could not participate, and fertile female study participants had to utilize a proper method of contraception. In the pre-study approved version of the protocol, inclusion criteria thresholds were set to >3·9 mg/L for C-reactive protein (CRP) and <27·5 kg/m^2^ for body mass index (BMI) (Weiser et al., 2021). New meta-analytic evidence published after the initiation of the study indicated that peripheral CRP may not be satisfyingly suited and sensitive to distinguish between those with and without low-grade inflammation affecting the brain in schizophrenia (Pillinger et al., 2019), and indeed very few otherwise eligible patients had CRP levels above the threshold, hence the CRP criterion was removed. Based on initial observations, the BMI threshold of 27·5 kg/m^2^ excluded a disproportionally large group of patients from participation beyond the theoretical justification. Accordingly, the threshold was increased to BMI <30·0 kg/m^2^. Both these protocol amendments were approved by the Regional Committee for Medical Research Ethics Western Norway in February 2019, and in addition by the Norwegian Medicines Agency. To ensure that the patient perspective was preserved, a reference group of individuals with lived experience was involved in the planning and implementation of the study. Both in- and outpatients could participate, but all study participants had to be in a treatment relationship with the psychiatrist referring them to the study.

### 2.3 Randomization and masking

Randomization was performed by the Research and Development department at Haukeland University Hospital, with block randomization stratified for sex and the different study centres. Treatment randomization codes were not available to the study staff assigning them to the trial groups. The randomization lists were shipped in a sealed opaque envelope to the study drug manufacturer (Kragerø tablettproduksjon AS, Norway) for preparation of prednisolone and placebo tablets with identical appearance. Medical doctors not participating in assessing the outcomes or analysing the data were recruited to monitor blood analyses weekly during the intervention phase.

### 2.4 Procedures

**Fig. 1:**
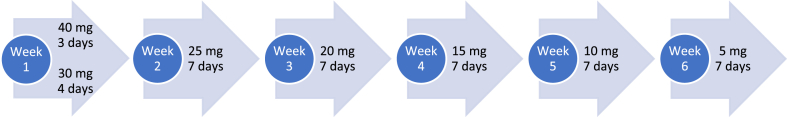
Dosing scheme of study medication (prednisolone).

Included participants were randomized to either prednisolone or placebo (1:1) and followed for 52 weeks after randomization. Prednisolone or placebo were added to existing treatment with antipsychotic medication. Concomitant use of other prescribed medications was not restricted. The study medication was 40 mg prednisolone daily for 3 days, followed by a tapering phase, whereas a control group received the placebo treatment for 6 weeks (see Fig. 1).

### 2.5 Assessments and precautions

The follow-up intervals after screening were at week 0 (baseline), weeks 1, 2, 3, 4, 5, 6 (end of intervention), week 26, and finally at week 52. Symptom severity of psychosis was measured with the PANSS, and the raters were all trained and certified by the PANSS Institute to secure inter-rater reliability. At each study visit, potential neuro-psychiatric side effects of prednisolone and possible adverse events were assessed. Overall functioning was measured with the Global Assessment of Functioning (GAF) scale – split version (Jones et al., 1995). Mental state was monitored repeatedly by the PANSS, the Calgary Depression Scale for Schizophrenia (CDSS) (Addington et al., 1992), and the Young Mania Rating Scale (YMRS) (Young et al., 1978).

Potential side effects were assessed with the UKU Side Effect Rating Scale (Lingjaerde et al., 1987), consisting of patient-rated answers to questions grouped primarily per psychiatric, neurological, and autonomic side effects. Safety data included fasting morning blood glucose, haemoglobin A1c (HbA1c), and leukocytes. The physicians evaluating the blood sample results against established reference areas (glucose: 4·0–6·0 mmol/L; HbA1c: ≤48 mmol/mol; leucocytes: 4·1–9·8 10^9^/L) were not involved in collecting any other study data from participants to maintain the blinding, as prednisolone may cause elevated levels of blood glucose and thereby compromise the masking. All participants received calcium and vitamin D supplementation in the treatment period as an extra precaution to prevent future bone fragility.

A data safety monitoring board evaluated the study progress. Clinical monitoring was applied, corresponding to the International Conference on Harmonization of Good Clinical Practice (ICH-GCP) standards by the Department of Research and Development, Haukeland University Hospital, Norway, for all three study centres.

### 2.6 Outcomes

The primary outcome was the difference in psychotic symptom severity between the prednisolone and placebo groups, expressed as a difference in the PANSS total score at the end of the 6-week treatment with either prednisolone or placebo. Clinically relevant secondary outcomes were a 6- and 12-month follow-up assessment of the PANSS total score, subscale differences in the PANSS, difference in overall functioning measured by GAF scores, severity of depression measured by the CDSS, and the measurement of various immunological markers and the aforementioned safety data.

### 2.7 Statistical analysis

The study design description and power calculation are also presented in greater detail in the protocol publication (Nasib et al., 2020). Based on the power calculation, the estimated total sample size was 90 participants. All analyses were performed with the statistical software R (R Core team, 2023). P-values below 5 % were considered statistically significant. The dataset was analysed longitudinally with linear mixed-effects (LME) models. In the analysis of the primary and secondary outcomes, treatment group, time, and the interaction between treatment group and time were included as independent variables. Patient ID was included as a random intercept to account for dependencies in the data due to repeated measurements. Sensitivity analyses were performed with age, sex, and baseline values of the dependent variable included as independent variables. Comparisons between the prednisolone and placebo group were analysed for all study visits throughout the year of follow-up. The study was registered in the European Union Clinical Trials Register (EudraCT no. 2017-000163-32).

### Role of the funding source

The funders did not take part in the study design, data collection, analysis and interpretation of data, or the writing of the manuscript. The study was independent of the pharmaceutical industry.

## 3 Results

**Fig. 2:**
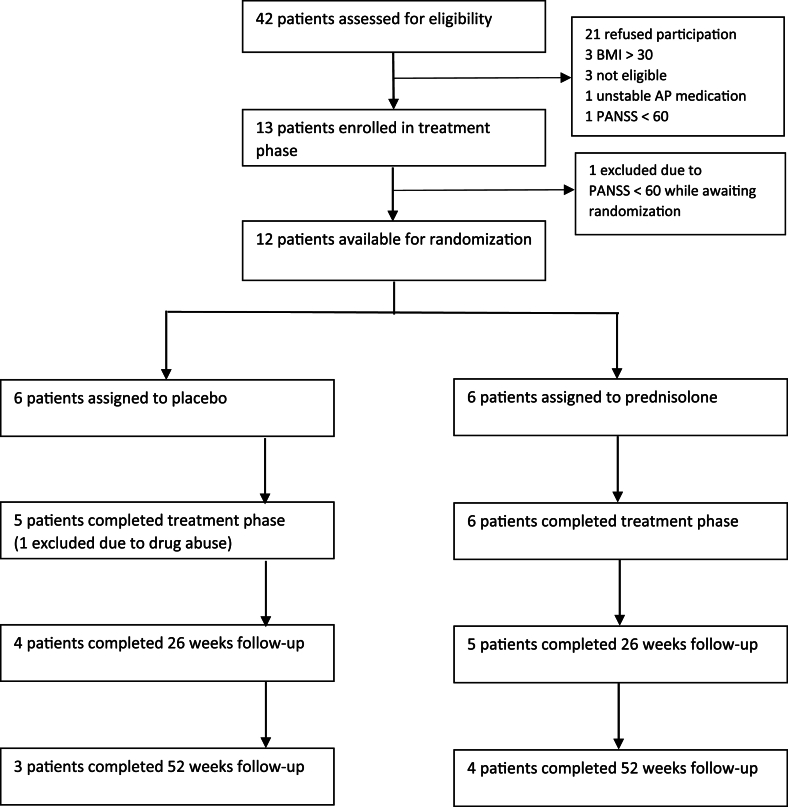
CONSORT flow diagram.

Thirteen patients with a schizophrenia diagnosis (DSM-IV: 295∗) were included across three sites between December 5, 2018 and January 25, 2022 (Fig. 2). One patient was excluded before randomization; thus 12 patients were included in the analyses: eight men and four women (see Table 1). Age ranges in prednisolone and placebo groups were 18–39 years and 21–34 years, respectively.

**Table 1:** Demographic and clinical characteristics at baseline.

	Prednisolone	Placebo	Group comparisons
	**n (%)**	**n (%)**	**p-value**
*N*	6 (50 %)	6 (50 %)	
*Male sex*	5 (83·3 %)	3 (50 %)	0·545
*Alcohol ≥ 4 times a week*	2 (33·3 %)	0 (0 %)	0·455
*Illicit drugs ≥ once a week*	1 (16·7 %)	1 (16·7 %)	1
*Inflammatory autoimmune disease∗*	1 (16·7 %)	0 (0 %)	1

	**mean (SD)**	**mean (SD)**	**p-value**
*Age*	29·8 (9·3)	28·5 (5·3)	0·774
*BMI*	26·0 (3·1)	22·8 (4·1)	0·154
*PANSS total*	82·3 (15·6)	84·5 (11·5)	0·791
*PANSS positive*	21·2 (6·0)	19·5 (3·9)	0·583
*PANSS negative*	24·3 (6·5)	24·2 (5·4)	0·963
*PANSS general*	36·8 (6·2)	40·8 (6·1)	0·285
*DUP (weeks)*	22·2 (22·8)	16·8 (9·6)	0·647
*Serum sodium (Na) (mmol/L)*	139·8 (1·8)	141.5 (0·8)	0·083
*Serum glucose (mmol/L*	5·1 (0·6)	5·0 (0·3)	0·777
*HbA1c (mmol/mol)*	33·5 (3·9)	31·0 (2·7)	0·244
*CRP (mg/L)*	3·5 (5·3)	0·8 (0·6)	0·268
*Leukocytes (10*^*9*^*/L)*	8·7 (1·5)	5·0 (0·8)	0·001
*CDSS*	3·7 (4·5)	7·0 (4·9)	0·316
*DDD (baseline)*	1·6 (0·8)	1·5 (0·4)	0·719
*GAF-f*	38·8 (8·5)	37·0 (6·8)	0·751
*GAF-s*	36·3 (12·5)	32·6 (5·5)	0·616

**Figure legend.** ∗Stable phase without systemic anti-inflammatory agents throughout the study. BMI: body mass index. CDSS: Calgary Depression Scale for Schizophrenia. CRP: C-reactive protein. DDD: defined daily dose for antipsychotic medications. DUP: Duration of untreated psychosis. GAF: Global Assessment of Functioning, f = function, s = symptom. PANSS: the Positive and Negative Syndrome Scale. SD: standard deviation. T-tests are used for continuous variables and Fisher's exact test is used for categorical variables.

When the World Health Organization (WHO) on March 11th, 2020, declared the novel coronavirus (Covid-19) outbreak a global pandemic, study inclusion was put on hold. We experienced no events that compromised patient safety due to the Covid-19 outbreak for the patients receiving study medication. Unfortunately, it proved very difficult to include new participants in the year after the Covid-19-induced break, hence the study was prematurely terminated.

**Fig. 3:**
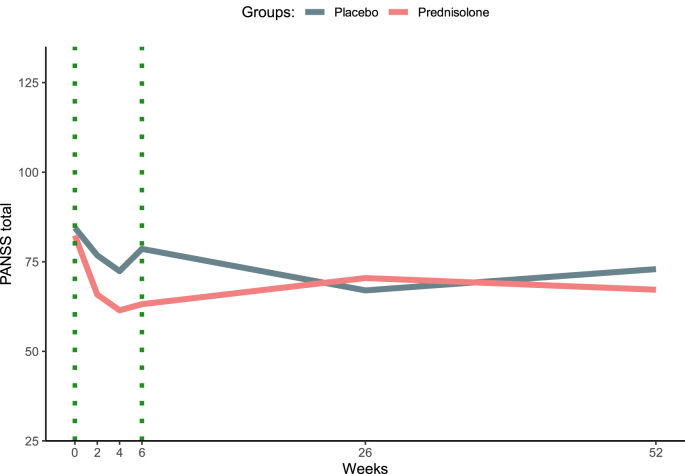
PANSS total score in the prednisolone group versus the placebo group (primary outcome) **Figure legend.** The vertical, dotted green lines indicate the start and end of prednisolone or placebo treatment.

The difference between treatment groups at various timepoints are model estimates given by the LME models. Regarding the primary outcome, the PANSS total score, the difference with standard deviation (SD) between the groups after 6 weeks was 15·4 (8·5), p-value 0·101. The symptom severity according to the PANSS total score was reduced from 82·3 at baseline to 63·2 after treatment in the prednisolone group (N = 6), compared to a reduction from 84·5 to 78·6 in the placebo group (N = 6), p-value 0·199. See Table 2 and Fig. 3.

**Fig. 4:**
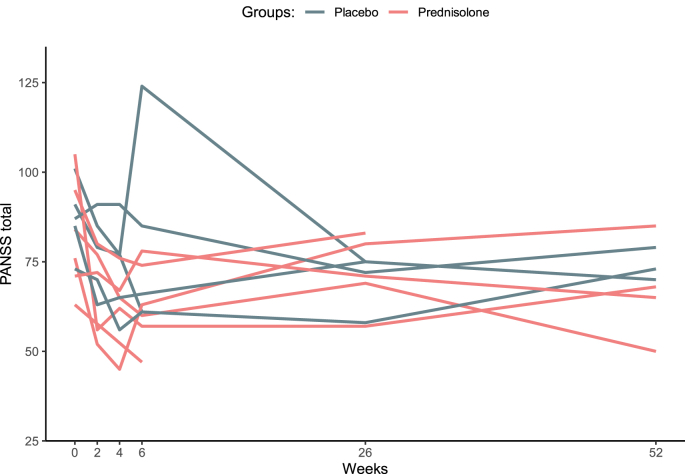
The courses of the individual PANSS scores during the year of follow-up.

Of note, one patient in the placebo group exhibited a different pattern than the majority (i.e., marked increase in PANSS total score at 6 weeks; Fig. 4). We therefore repeated the statistical procedure without this patient, as a sensitivity analysis. The overall results remained the same in the sensitivity analysis: The prednisolone group had a steeper fall in the PANSS total score compared to placebo. See Fig. IV in the Appendix.

**Fig. 5:**
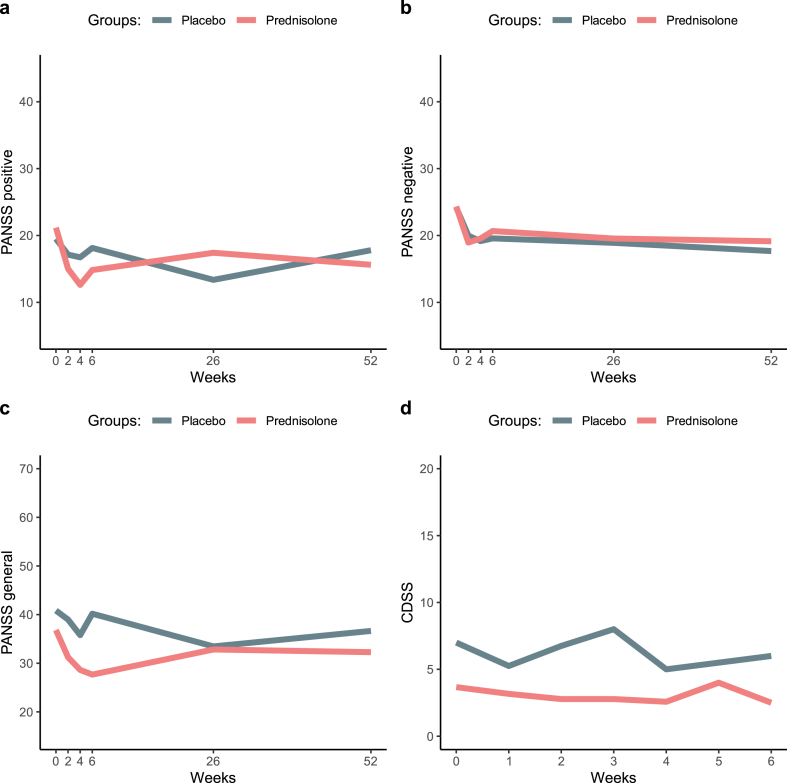
PANSS subscales (positive, negative, general) and CDSS for the prednisolone and placebo groups.

A similar pattern to the primary outcome was found for the secondary outcome PANSS positive score, where symptom difference between the groups showed a larger reduction in psychotic symptoms for the prednisolone group, however without reaching a statistically significant difference between the groups at week 6. See Fig. 5 and Table 2. For the PANSS negative score, there was overall no difference between the groups. For the PANSS general score, we observed a statistically significant difference (p = 0·021) between the prednisolone and the placebo group at week 6 (Table 2). Severity of depression, as measured with the CDSS (Addington et al., 1993), showed no significant differences between the prednisolone and placebo groups throughout the study period.

**Table 2:** Primary outcome (PANSS reduction).

		Baseline	2 weeks	4 weeks	6 weeks	26 weeks	52 weeks
PANSS total	Placebo	84·5 (5·8)	76·8 (6·3)	72·4 (6·3)	78·6 (6·3)	67·0 (6·9)	72·9 (7·8)
	Prednisolone	82·3 (5·8)	65·9 (6·3)	61·5 (6·3)	63·2 (5·8)	70·5 (6·3)	67·2 (6·9)
	Difference (SD) [p-value]	2·2 (8·2) [0·796]	10·9 (8·9) [0·246]	10·9 (8·9) [0·246]	15·4 (8·5) [0·101]	−3·4 (9·3) [0·720]	5·7 (10·4) [0·594]

PANSS positive	Placebo	19·5 (2·2)	17·1 (2·3)	16·7 (2·3)	18·1 (2·3)	13·4 (2·6)	17·8 (2·9)
	Prednisolone	21·2 (2·2)	15·0 (2·3)	12·6 (2·3)	14·8 (2·2)	17·4 (2·3)	15·6 (2·5)
	Difference (SD) [p-value]	−1·7 (3.1) [0·598]	2·1 (3·3) [0·536]	4·1 (3·3) [0·241]	3·3 (3·2) [0·325]	−4·0 (3·5) [0·269]	2·2 (3·8) [0·582]

PANSS negative	Placebo	24·2 (2·3)	20·0 (2·4)	19·2 (2·4)	19·6 (2·4)	18·9 (2·6)	17·7 (2.8)
	Prednisolone	24·3 (2·3)	18·9 (2·4)	19·5 (2·4)	20·7 (2·3)	19·5 (2·4)	19·1 (2·6)
	Difference (SD) [p-value]	−0·2 (3·3) [0·960]	1·0 (3·4) [0·772]	−0·4 (3·4) [0·914]	−1·1 (3·3) [0·749]	−0·6 (3·5) [0·858]	−1·5 (3·8) [0·706]

PANSS general	Placebo	40·8 (3.1)	39·0 (3·4)	35·8 (3·4)	40·2 (3·4)	33·5 (3·7)	36·6 (4·1)
	Prednisolone	36·8 (3·1)	31·2 (3·4)	28·6 (3·4)	27·7 (3·1)	32·8 (3·4)	32·3 (3·6)
	Difference (SD) [p-value]	4·0 (4·4) [0·385]	7·8 (4·8) [0·134]	7·2 (4·8) [0·163]	12·5 (4·6) [0·021]	0·6 (5·0) [0·901]	4·4 (55·0) [0·446]

**Figure legend.** All numbers were estimated with the LME model described in the statistics section. The difference was calculated by subtracting the PANSS level in the prednisolone group from the PANSS level in the placebo group at each time point. SD: standard deviation.

No carry-over effects of treatment with the study drug were detected beyond 6 weeks; at 6 and 12 months, the PANSS trajectories aligned to parallels for prednisolone and placebo. The two groups were comparable per the use of benzodiazepines, antidepressants, and mood stabilizers (lithium and anticonvulsants). Analyses without the one patient in the prednisolone group with a comorbid autoimmune inflammatory disease did not affect the findings (see Supplementary Figure III).

Regarding safety, none of the participants randomized to prednisolone experienced a worsening of psychotic symptoms during the initial 6 weeks when the study drug was administered. Similarly, no patients exhibited a worsening of depression (Fig. 5). No neuro-psychiatric side effects related to prednisolone were recorded. Specifically, none of the patients developed a manic episode, and there was no difference in the YMRS between groups.

We recorded glucose, HbA1c, and leukocytes and sodium (Na) in the blood, monitoring possible side effects and indirectly documenting compliance with study medication. See Fig. 6 in Appendix: No patient exceeded the defined blood glucose upper level of 11 mmol/L, although the level of fasting blood glucose did increase as expected in the prednisolone group. This effect was also reflected in the elevated level of HbA1c at week 26 (diff. = 5·2, p-value = 0·052) and week 52 (diff. = 5·9, p-value = 0·049). The prednisolone group had an increase in leukocytes (Supplementary Figures A–F in the Appendix) during the study intervention phase, as expected due to the pharmacological properties of prednisolone.

The average CRP level in the prednisolone group was 3.5 (SD 5.3) and the level in the placebo group was 0.8 (SD 0.6). Although this did not represent a statistically significant difference (p-value 0.268), we investigated the CRP-levels at an individual level and found that the difference was mainly due to one outlier with a high CRP (Gangadin et al., 2024). When the outlier was left out of the analyses, the averages were more comparable and the PANSS total score results were essentially unchanged (see Fig. III in Appendix).

Three serious adverse events (SAEs) were reported in the placebo group, compared to none in the prednisolone group. The SAEs included one strangulation attempt without somatic consequence, one hospital admission to a somatic ward to rule out a possible cardiac event, and one hospital admission due to exacerbation of psychosis. None of these incidents were considered to be related to study medication or participation in the NorPEPS. When the patient-rated side effect scores (UKU) were compared, they were in favour of the prednisolone group for all subcategories. See Table A in the Appendix.

## 4 Discussion

This proof-of-concept pilot study indicated that a prednisolone add-on was safe, well tolerated, and potentially effective at reducing symptoms of psychosis in patients with schizophrenia who used stable antipsychotic medication. The study medication was administered for a 6-week period, during which the medication was tapered off and stopped. The difference between the prednisolone- and placebo-treated groups did not reach statistical significance for the primary outcome (PANSS total score) after 6 weeks, but we observed a statistically significant difference in the PANSS general score and a nonsignificant trend for the PANSS total score at this point.

Prednisolone has the potential to trigger psychosis and mood disturbances, also when used correctly, although the mechanisms remain unclear (Holler et al., 1963; Huynh and Reinert, 2021). Thus, monitoring of the included patients’ mental state was crucial. However, no exacerbations of mood symptoms or suicidality were present in the prednisolone group. One patient in the prednisolone group who due to symptoms of a cold ended the add-on treatment 2 weeks earlier had a non-significant increase in the PANSS score at the following visit. The reasons for this could be the infection itself, the psychotic disorder, an effect of prednisolone, or other unknown factors. No other patients in the prednisolone group had an increase in psychotic symptoms that could be attributed to prednisolone or study participation. All SAEs occurred in the placebo group and were regarded as independent of the study, and the patient-rated side effect scores (UKU) were in favour of the prednisolone group. A similar, benign safety profile was found in the study by Nasib et al. (2021), encouraging future investigations of prednisolone in larger samples of patients with schizophrenia. Safety and tolerability were found to be manageable when prednisolone was combined with antipsychotics, even in the symptomatic phase of schizophrenia. Finally, at week 26, 4 of 6 patients completed the visit in the placebo group compared to 5 of 6 patients in the prednisolone group.

Despite the nonsignificant difference in PANSS total between the prednisolone and placebo groups, the data may be interpreted as a signal of potential beneficial effects of prednisolone as an add-on treatment for schizophrenia. Lending support to the results are the fact that all six patients treated with prednisolone showed the same pattern of steep initial symptom reduction during the 6 weeks of treatment, as compared to the six placebo-exposed patients. Furthermore, there seemed to be a dose–response pattern, with the steepest reduction of psychosis symptoms corresponding to the highest prednisolone dosages, followed by a flattening of the response curves at the lowest dosages, and finally slightly increasing symptoms of psychosis after discontinuation of prednisolone. One participant in the prednisolone group was, after consulting with the Data Safety Monitoring Board, urged to discontinue the study medication prematurely due to suspected Covid-19 infection. The premature discontinuation of prednisolone was followed by an earlier increase of psychosis symptoms compared to the rest of the prednisolone group, lending further support to a dose-response relationship. Finally, the findings correspond with extensive evidence of involvement of inflammation in schizophrenia.

Prednisolone has potential long-term metabolic effects that may be problematic for this patient population, i.e. diabetes, weight gain, and possibly increased risk of cardiovascular disease. Therefore, a 6-week treatment period with doses tapered from 40 mg to 0 was chosen after balancing the risks and benefits of prednisolone. This titration schedule was chosen after consultation with specialists in internal medicine, and the treatment guideline for the exacerbation of active phase inflammatory bowel disease was adopted (Nasib et al., 2020). However, the HbA1c, reflecting the blood glucose levels from the past 2–3 months, was also elevated in the prednisolone group after 6 months and 12 months compared to the placebo group, reaching statistical significance at week 52. Accordingly, this was unlikely to be a prednisolone effect, but it might have been associated with the antipsychotic medication or other factors.

Few placebo-controlled studies have examined the effects of corticosteroid medicines on symptom severity in schizophrenia, but as mentioned in the Introduction, Nasib et al., who applied a comparable research protocol, found no effect of prednisolone in their sample of 42 patients randomized to prednisolone or placebo (Nasib et al., 2021). Reasons for this apparent discrepancy may include that our patient sample was diagnostically more homogenous, given that all participants in NorPEPS had a DSM-IV 295∗ or F20 schizophrenia diagnosis in the ICD-10. In addition, the percentage of patients with clozapine treatment and older age at baseline was higher in the prednisolone group in the study conducted by Nasib et al., possibly indicative of a more treatment-resistant patient sample. Anti-inflammatory agents have been postulated to be more effective at an early stage in schizophrenia development, preferably given during the first episode of psychosis (FEP) or even to high-risk populations (Girgis et al., 2018; Khandaker et al., 2018).

We cannot rule out that results may have been affected by considerations made during inclusion, or by signs of heterogeneity in the study groups. CRP levels and BMI displayed different group level at baseline, although not statistically significantly. Although one should be careful drawing too firm conclusions from a pilot study, based on the interesting – and statistically significant – finding of higher leukocytes levels in the prednisolone group prior to the intervention, it cannot be ruled out that this could represent a partial explanation for the findings.

Although we did not use any specific inflammation-related parameter to select our study sample, we still think that it is probable that an anti-inflammation treatment may be more efficacious in a sample selected by an inflammation-related marker. However, it is for now not clear what would be the best marker to select a high-inflamed sample in future studies, and it is conceivable that such a marker should be specific for the treatment at trial, for example that trials of tocilizumab, the interleukin-6-receptor antibody, should use a high level of IL-6 as a selection criterion for the study sample (Girgis et al., 2018). As prednisolone has broad mechanisms of action, and no other baseline characteristic was statistically significant in our sample, we cannot point to possible novel selection criteria for further prednisolone trials in schizophrenia based on the results from this study.

The selected BMI threshold of 30·0 kg/m^2^ could have excluded a significant proportion of participants with immune-metabolic dysfunction. However, since prednisolone has the potential to induce diabetes mellitus, and safety was considered crucial in this setting, with a study population vulnerable to metabolic abnormalities, this threshold was chosen (Balotsev et al., 2017). The 7-year timeframe from symptom onset until study inclusion could have contributed to heterogeneity in the sample, whereas the fact that all participants were diagnosed with F20 Schizophrenia should ensure sample homogeneity. The average duration of untreated psychosis (DUP) was 22.2 weeks (SD 22.8) in the prednisolone group and 16.8 weeks (SD 9.6) in the placebo group, with no statistically significant difference between the groups (p-value 0.647).

Another point is that the patient with the high CRP (14 mg/L) is the same individual who has an inflammatory autoimmune disease. This inflammatory autoimmune disease was however in a stable phase with no exacerbations or need for extra treatment during the entire study participation. If not, the participant would have been excluded. It is possible that this individual is having a slightly higher habitual level of inflammation. Reanalyses without data from the participant did not change the results.

The strength of the study is not in its number of included patients, but in its thorough, comprehensive medical assessments and robust double-blind design. Due to Covid-19-related restrictions on patient inclusion, the study became markedly underpowered, and the results require replication. Also, investigation of potential long-term effects like cardio-vascular and metabolic risk increases must be further investigated in future studies of prednisolone, due to both the pharmacological properties of prednisolone and the patient population with an increased risk of cardiovascular and metabolic comorbidities. Nevertheless, our results provide a clear incentive to pursue further investigation to confirm findings and understand the underlying mechanisms of action in order to evolve the treatment of psychosis.

## CRediT authorship contribution statement

**Gunnhild E. Hoprekstad:** Writing – review & editing, Writing – original draft, Visualization, Validation, Resources, Project administration, Methodology, Investigation, Formal analysis. **Erik Johnsen:** Writing – review & editing, Writing – original draft, Visualization, Validation, Supervision, Resources, Project administration, Methodology, Investigation, Funding acquisition, Formal analysis, Conceptualization. **Christoffer Bartz-Johannessen:** Writing – review & editing, Supervision, Software, Methodology, Formal analysis, Data curation. **Jill Kristin Bjarke:** Writing – review & editing, Methodology, Investigation, Conceptualization. **Petros Drosos:** Writing – review & editing, Investigation. **Ole Bernt Fasmer:** Writing – review & editing, Conceptualization. **Kristian Varden Gjerde:** Writing – review & editing, Investigation. **Solveig K. Reitan:** Writing – review & editing, Supervision, Project administration, Methodology, Investigation, Formal analysis, Conceptualization. **Silje Skrede:** Writing – review & editing, Validation, Supervision, Methodology, Investigation, Conceptualization. **Iris E.C. Sommer:** Writing – review & editing, Validation, Supervision, Methodology, Data curation. **Lena Stabell:** Writing – review & editing, Validation, Software, Project administration, Investigation, Data curation. **Vidar M. Steen:** Writing – review & editing, Validation, Supervision, Conceptualization. **Rune A. Kroken:** Writing – review & editing, Validation, Supervision, Project administration, Methodology, Investigation, Conceptualization.

## Data sharing

According to Norwegian law, data sharing requires approvals from the Regional Committees for Medical and Health Research Ethics, and from the Data Protection Officer at Haukeland University Hospital, Bergen, based on specific research proposals.

## Funding

This work was supported by the Western Norway Regional Health Authority (#F-11490-91221, #912163), in addition to the universities and hospitals. We thank the study participants, data collectors, medical monitors, the principal investigator at Stavanger, and the clinical advisors at the three study sites for their essential contributions.

## Declaration of competing interest

The authors declare the following financial interests/personal relationships which may be considered as potential competing interests: Gunnhild Hoprekstad and Erik Johnsen reports financial support was provided by Western Norway Regional Health Authority. Iris E. C. Sommer reports a relationship with Gabather that includes: consulting or advisory. Iris E. C. Sommer reports a relationship with Otsuka that includes: consulting or advisory. Iris E. C. Sommer reports a relationship with 10.13039/501100013327Lundbeck that includes: funding grants. Iris E. C. Sommer reports a relationship with Janssen that includes: funding grants. Petros Drosos reports a relationship with Western Norway Regional Health Authority that includes: funding grants. Rune A. Kroken reports a relationship with Western Norway Regional Health Authority that includes: funding grants. If there are other authors, they declare that they have no known competing financial interests or personal relationships that could have appeared to influence the work reported in this paper.

## Data Availability

The data that has been used is confidential.
