# Supplementary material for: Prednisolone add-on in early phase schizophrenia: A randomized, double-blind, placebo-controlled pilot study
Source: Brain Behav Immun Health. 2025 Jul 10;48:101047. doi: 10.1016/j.bbih.2025.101047 (PMC12302662; doi:10.1016/j.bbih.2025.101047)
Supplement: Multimedia component 1 [file mmc1.docx]

**Appendix**

1. Protocol
2. Supplementary figures: fasting blood glucose, HbA1c, and sensitivity analyses I-V
3. CRP
4. Table A: Patient-rated side effects (UKU)
5. Impact of the Covid-19 pandemic on enrolment
6. **Protocol**

The Norwegian Prednisolone in Early Psychosis Study - NorPEPS

The role of immune-modulating strategies in the treatment of psychosis

Version 3.0

26.06.2017

**PROTOCOL TITLE:** The Norwegian Prednisolone in Early Psychosis Study - NorPEPS: the role of immune-modulating strategies in the treatment of psychosis

| **Protocol ID** |  |
| --- | --- |
| **Short title** | **NorPEPS** |
| **EudraCT number** | 2017-000163-32 |
| **Version** | **3.0** |
| **Date** | **26.06.2017** |
| **Coordinating investigator/project leader** | **Prof. Erik Johnsen, MD, PhD**  erik.johnsen@helse-bergen.no  Haukeland University Hospital  Division of Psychiatry  Phone: +4792456225 |
| **Principal investigator Bergen** | **Prof. Erik Johnsen, MD, PhD**  erik.johnsen@helse-bergen.no  Haukeland University Hospital  Division of Psychiatry  Phone: +4792456225 |
| **Deputy coordinating investigator/ Sub investigator Bergen** | **Assoc. Prof. Rune A. Kroken, MD, PhD**  rune.andreas.kroken@helse-bergen.no  Haukeland University Hospital  Division of Psychiatry  Phone: +4792095205 |
| **Principal investigator Trondheim** | **Prof. Arne Vaaler, MD, PhD**  [arne.e.vaaler@ntnu.no](mailto:arne.e.vaaler@ntnu.no)  St Olavs Hospital  Division of Psychiatry  Phone: +4792090415 |
| **Principal investigator Stavanger** | **Assoc. Prof. Helle Schøyen, MD, PhD**  [helle.kristine.schoyen@sus.no](mailto:helle.kristine.schoyen@sus.no)  Stavanger University Hospital  Division of Psychiatry  Phone: +92451169 |
| **Sponsor** | Haukeland University Hospital  Division of Psychiatry  Att: Director Hans Olav Instefjord  [hans.olav.instefjord@helse-bergen.no](mailto:hans.olav.instefjord@helse-bergen.no)  Phone: +4792456225 |
| **Subsidising party** | **The Western Norway Regional Health Authority** |
| **Independent expert** |  |
| **Laboratory sites** | Haukeland University Hospital/ St. Olavs Hospital/ Stavanger University Hospital  Laboratory for Clinical Chemistry/ Hormone laboratory  Phone: +47 55975000 |
| **Pharmacy** | *Production of study medication:*  **Kragerø Tablettproduksjon AS** Anne Hopstock, MPharm, QP Clinical studies  annehops@hotmail.com  Phone: +4791322546 |
| **Study monitor** |  |

**PROTOCOL SIGNATURE SHEET**

| **Name** | **Signature** | **Date** |
| --- | --- | --- |
|  |  |  |
| **[Coordinating Investigator/Project leader/Principal Investigator]:**  **Prof. Erik Johnsen, MD, PhD** |  |  |

| **Name** | **Signature** | **Date** |
| --- | --- | --- |
|  |  |  |
| **[Principal Investigator St. Olav Hospital]:**  **Prof. Arne Vaaler, MD, PhD** |  |  |

| **Name** | **Signature** | **Date** |
| --- | --- | --- |
|  |  |  |
| **[Principal Investigator Stavanger University Hospital]:**  **Assoc. Prof. Helle Schøyen, MD, PhD** |  |  |

**TABLE OF CONTENTS**

[1. SUMMARY 8](#_Toc360716183)

[2. INTRODUCTION AND RATIONALE 10](#_Toc360716184)

[3. OBJECTIVES 15](#_Toc360716185)

[4. STUDY DESIGN 16](#_Toc360716186)

[5. STUDY POPULATION 18](#_Toc360716187)

[5.1 Population 18](#_Toc360716188)

[5.2 Inclusion criteria 18](#_Toc360716189)

[5.3 Exclusion criteria 18](#_Toc360716190)

[5.4 Sample size calculation 19](#_Toc360716191)

[6. TREATMENT OF SUBJECTS 21](#_Toc360716192)

[6.1 Investigational product/treatment 21](#_Toc360716193)

[6.2 Use of co-intervention (if applicable) 21](#_Toc360716194)

[6.3 Escape medication (if applicable) 21](#_Toc360716195)

[7. INVESTIGATIONAL PRODUCT 22](#_Toc360716196)

[7.1 Name and description of investigational product(s) 22](#_Toc360716197)

[7.2 Summary of findings from non-clinical studies 22](#_Toc360716198)

[7.3 Summary of findings from clinical studies 22](#_Toc360716199)

[7.4 Summary of known and potential risks and benefits 22](#_Toc360716200)

[7.5 Description and justification of route of administration and dosage 24](#_Toc360716201)

[7.6 Dosages, dosage modifications and method of administration 24](#_Toc360716202)

[7.7 Preparation and labelling of Investigational Medicinal Product 25](#_Toc360716203)

[7.8 Drug accountability 25](#_Toc360716204)

[8. METHODS 2](#_Toc360716205)7

8.[1 Study parameters/endpoints 2](#_Toc360716206)7

[8.1.1 Main study parameter/endpoint 2](#_Toc360716207)7

[8.1.2 Secondary study parameters/endpoints (if applicable) 2](#_Toc360716208)7

8.[2 Randomisation, blinding and treatment allocation 2](#_Toc360716209)7

[8.2.1 Randomization 2](#_Toc360716210)7

[8.2.2 Unblinding Procedure 2](#_Toc360716211)8

[8.3 Study procedures 2](#_Toc360716212)8

[8.4 Withdrawal of individual subjects 3](#_Toc360716213)5

[8.5 Replacement of individual subjects after withdrawal 3](#_Toc360716214)6

[8.6 Follow-up of subjects withdrawn from treatment 3](#_Toc360716215)6

[8.7 Premature termination of the study 3](#_Toc360716216)6

[9. SAFETY REPORTING 3](#_Toc360716217)7

[9.1 Section 10 WMO event 3](#_Toc360716218)7

[9.2 AEs, SAEs and SUSARs 3](#_Toc360716219)7

[Adverse events (AEs) 3](#_Toc360716220)7

[Serious adverse events (SAEs) 3](#_Toc360716221)7

[Suspected unexpected serious adverse reactions (SUSARs) 3](#_Toc360716222)8

[9.3 Annual safety report 3](#_Toc360716223)8

[9.4 Follow-up of adverse events 3](#_Toc360716224)9

[9.5 Data Safety Monitoring Board (DSMB) 3](#_Toc360716225)9

[10. STATISTICAL ANALYSIS 40](#_Toc360716226)

[10.1 Primary study parameter(s)](#_Toc360716227) 40

[10.2 Secondary study parameter(s)](#_Toc360716228) 40

[10.3 Other study parameters 4](#_Toc360716229)1

[10.4 Prediction model for response to prednisolone 4](#_Toc360716230)1

[11. ETHICAL CONSIDERATIONS 4](#_Toc360716231)2

[11.1 Regulation statement 4](#_Toc360716232)2

[11.2 Recruitment and consent 4](#_Toc360716233)2

[11.3 Benefits and risks assessment, group relatedness 4](#_Toc360716234)2

[11.4 Compensation for injury 4](#_Toc360716235)3

[11.5 Incentives (if applicable) 4](#_Toc360716236)4

[12. *A*DMINISTRATIVE ASPECTS, MONITORING AND PUBLICATION 4](#_Toc360716237)5

[12.1 Handling and storage of data and documents 4](#_Toc360716238)5

[12.2 Monitoring and Quality Assurance 4](#_Toc360716239)5

[12.3 Amendments 4](#_Toc360716240)6

[12.4 Annual progress report 4](#_Toc360716241)6

[12.5 End of study report 4](#_Toc360716242)6

[12.6 Public disclosure and publication policy 4](#_Toc360716243)7

[13. STRUCTURED RISK ANALYSIS 4](#_Toc360716244)8

[13.1Potential issues of concern 4](#_Toc360716245)8

[13.2Synthesis 5](#_Toc360716246)4

[13.3 Stopping rules – individual participants 5](#_Toc360716247)5

13.4 Stopping rules - study as a whole 55

[14. REFERENCES 5](#_Toc360716248)6

**LIST OF ABBREVIATIONS AND RELEVANT DEFINITIONS**

| **ABR** | **ABR form, General Assessment and Registration form, is the application form that is required for submission to the accredited Ethics Committee** |
| --- | --- |
| **AE** | **Adverse Event** |
| **AR** | **Adverse Reaction** |
| **CA** | **Competent Authority** |
| **CCMO** | **Central Committee on Research Involving Human Subjects** |
| **CV** | **Curriculum Vitae** |
| **DSMB** | **Data Safety Monitoring Board** |
| **EU** | **European Union** |
| **EudraCT** | **European drug regulatory affairs Clinical Trials** |
| **GCP** | **Good Clinical Practice** |
| **IB** | **Investigator’s Brochure** |
| **IC** | **Informed Consent** |
| **IMP** | **Investigational Medicinal Product** |
| **IMPD** | **Investigational Medicinal Product Dossier** |
| **METC** | **Medical research ethics committee (MREC)** |
| **(S)AE** | **(Serious) Adverse Event** |
| **SPC** | **Summary of Product Characteristics** |
| **Sponsor** | **The sponsor is the party that commissions the organisation or performance of the research, for example a pharmaceutical**  **company, academic hospital, scientific organisation or investigator. A party that provides funding for a study but does not commission it is not regarded as the sponsor, but referred to as a subsidising party.** |
| **SUSAR** | **Suspected Unexpected Serious Adverse Reaction** |
| **Wbp** | **Personal Data Protection Act** |
| **WMO** | **Medical Research Involving Human Subjects Act** |

# SUMMARY

**Rationale:** There is ample evidence that inflammatory processes in the central nervous system play a role in the pathophysiology of schizophrenia. Considering the need for new treatment options, anti-inflammatory drugs can be viewed as potential candidates to improve schizophrenia outcome, as modulation of the immune response in the brain could prevent grey matter loss and the associated increase in negative symptoms and cognitive deficits. Several immune-modulating agents such as NSAIDs, minocycline and statins have already been studied, however they have a low potency and so far only a modest effect was shown (Orr, 2008, Reiss and Wirkowski, 2009). Corticosteroids are effective in various chronic inflammatory and auto-immune disorders (Lowenberg et al., 2008), with a positive effect on cognition in multiple sclerosis (Zephir et al., 2005, Zephir et al., 2008). From the group of glucocorticosteroids, prednisolone has minor mineral-corticosteroid potencies, can adequately pass the blood-brain-barrier and its side effects and safety profile are well known. Therefore, treatment with prednisolone can be used as a proof of concept to investigate the possibility of immune modulation as a treatment for schizophrenia. Screening on a broad inflammation marker, C-reactive protein (CRP), will be performed and patients will be selected based on their CRP level. Elevated plasma CRP levels are associated with a higher risk of (late onset) schizophrenia (Wium-Andersen et al., 2014). An elevated level of serum CRP has found to be strongly associated with cognitive dysfunction in schizophrenia (Dickerson et al., 2013, Johnsen et al., 2016). Previous studies have shown an enhanced symptom reducing effect of adjunctive immunomodulating medicine in antipsychotic treated patients with schizophrenia, and depression-lowering abilities in psoriasis patients. (Tyring et al., 2006, Laan et al., 2010).

Hypothesis: Daily treatment with prednisolone in addition to antipsychotic treatment reduces psychotic symptoms and improves cognition, as compared to placebo.

**Objective**: The primary objective of this trial is to investigate whether prednisolone improves symptom severity as compared to placebo when given in addition to antipsychotic medication to patients with early-stage psychotic disorder. Secondary objectives include improvement of cognitive functioning and positive, negative and general psychopathological symptoms as well as general functioning.

**Study design:** Randomized placebo-controlled double-blind trial.

**Study population:** 90 men and women, with an age of 18-70 years, diagnosed with schizophrenia, schizoaffective or schizophreniform disorder (DSM-IV 295.*) or psychosis NOS (not otherwise specified) (298.9). The time interval between the onset of psychosis and study entry should not exceed five years and CRP level should be at least 3.9 mg/L.

**Intervention**: Patients will be randomized 1:1 to either prednisolone or placebo daily for a period of 6 weeks. Identical tablets will be administered. Prednisolone will be initiated at 40 mg for three days, after which it will be phased out within 6 weeks after start, following current treatment guidelines.

**Main study parameters/endpoints:** Primary outcome is change in symptom severity, expressed as a change in total score on the Positive and Negative Syndrome Scale (PANSS) from baseline to end of the 6-week treatment. Secondary outcomes are a 6-month follow-up assessment of PANSS, cognitive functioning (measured through the Brief Assessment of Cognition in Schizophrenia; BACS), change in GAF scores and the measurement of various immunological biomarkers. In post-hoc analyses, we will attempt to identify baseline blood markers with predictive properties regarding improvement in the prednisolone treatment arm.

**Nature and extent of the burden and risks associated with participation, benefit and group relatedness:** Use of prednisolone is associated with a certain risk of side effects.

Patients will only be included if they are overall physically healthy, and relevant health aspects are monitored throughout the study. During the treatment period, patients will be interviewed at weekly intervals to assess symptom severity, mood, global functioning, side effects and suicidal ideation. Blood will be drawn at five occasions with negligible and known risks (e.g. irritation). Patients will have follow-up visits at 6 and 12 months after the end of treatment to assess symptom severity, cognition, global functioning and immune parameters. We expect a decrease in symptom severity, as low grade brain inflammation may be associated with psychotic symptoms. The burden and risks are acceptable taking into account that the results may give raise to a new line of scientific research as well as treatment options for a disabling disorder.

# INTRODUCTION AND RATIONALE

Schizophrenia is a severe mental disorder with a worldwide prevalence of around 1%, placing significant burden on global health (WHO, 2001). Although the introduction of antipsychotic medications in the 1950s has substantially improved clinical symptoms of schizophrenia (Tandon et al., 2010), the disease is still causing considerable morbidity and mortality (Saha et al., 2007). Patients are affected by substantial impairment in multiple domains of life. Psychosocial functioning is often significantly impaired, which is only partially explained by clinical symptom severity (Robertson et al., 2013). Competitive employment rates in schizophrenia are low compared with the general population (McGurk et al., 2009). Of patients with schizophrenia, 80% are unable to return to work following the first episode of psychosis; even when paid employment is achieved, job tenure is frequently a problem for patients, who appear to fare worse in the workforce than patients with other mental disorders (Kitchen et al., 2012). As many as two-thirds of people with schizophrenia are unable to fulfill basic social roles, such as spouse, parent, and employee, even when psychotic symptoms are in remission (Bellack et al., 2007). Also Agerbo et al. (Agerbo et al., 2004) found a strong long-term association between schizophrenia, singleness, disadvantaged socioeconomic position, and labor market marginalization, relatively unaffected by admission to a psychiatric hospital.

One factor underlying these impairments and difficulties are cognitive deficits; although not yet included in the diagnostic criteria for schizophrenia, they are considered a core feature of schizophrenia symptomatology (Kitchen et al., 2012). Cognitive impairments are the most consistent determinant of deficits in everyday functioning in schizophrenia (Green, 1996, Green et al., 2000). A recent review article by Kahn and Keefe (Kahn and Keefe, 2013) states that cognitive underperformance constitutes a (genetic) risk factor, precedes the onset of psychosis by many years, may continue to worsen after psychosis is established and determines outcome.

Although the efficacy of currently available antipsychotic treatment on positive symptoms is convincing, the effects on cognition in schizophrenia and first episode psychosis appear to be minimal and controversial (Keefe and Harvey, 2012). A meta-analysis by Mishara et.al. (Mishara and Goldberg, 2004) studying effects of typical antipsychotics on cognition found only modest effects, with a mean effect size of 0.22. More recent studies found comparable small effect sizes, comparing typical and atypical antipsychotics in long-term patients (Keefe et al., 2007) and in first episode patients (Davidson et al., 2009).

Given the large impact of the illness, the need for better treatment of patients with schizophrenia is high. However, development of new treatment paradigms is hampered by insufficient knowledge of the underlying disease mechanisms. The pathogenesis of schizophrenia is still far from elucidated. Different lines of evidence now suggest that low grade inflammation in the central nervous system is involved in the pathogenesis of schizophrenia. These include the altered risk of schizophrenia patients and their relatives for specific auto-immune diseases (Benros et al., 2011), clinical similarities between the course of schizophrenia and auto-immune disease (Knight et al., 2007) and decreased prevalence of schizophrenia in men who have used Non-Steroidal Anti-Inflammatory Drugs (NSAIDs) (Laan et al., 2007) for somatic disorders. Furthermore, an infectious cause or trigger is suggested by the observed association between schizophrenia and pre- and perinatal infections (Brown and Derkits, 2010), as well as by seroconversion to certain pathogens in patients with schizophrenia (Torrey et al., 2007). On a cellular level, inflammation of the central nervous system is suggested by an increased number of activated microglia cells in the brains of patients with schizophrenia as visualized by positron electron tomography (van Berckel et al., 2008, Doorduin et al., 2009). In an activated state, microglia cells can produce free radicals, pro-inflammatory components and other neurotoxic substances, causing cell death in their proximity (O'Donnell, 2012). The activation of microglia cells provides a possible route by which an inflammatory state in the brain could cause increased gray matter loss and consequently contribute to more severe negative and cognitive symptoms.

Indirectly supporting this line of thought, a randomized controlled trial by Raison et al. (Raison et al., 2013) on anti-TNF-alpha biological infliximab in treatment resistant depression found that, although no generalized efficacy of TNF antagonism was found, there was a significant decrease of symptoms (P = 0.01) in patients with an increased baseline level of C-reactive protein (CRP), an inflammatory parameter in blood. A treatment response of 62% was seen in patients with a CRP >5mg/L compared to 33% in the placebo-arm. Fan et al (Fan et al., 2007) found an association between elevated CRP levels and severity of psychotic symptoms. Moreover four cross-sectional studies on schizophrenia showed a negative correlation between CRP and cognitive functioning in people with schizophrenia (Dickerson et al., 2007, Dickerson et al., 2013, Dickerson et al., 2012, Johnsen et al., 2016). Also in a prospective study by Wium-Andersen et al. (Wium-Andersen et al., 2014) a baseline level of elevated plasma CRP was associated with a 6 to 11 fold increased risk of late and very late onset schizophrenia in the general population. Individuals with versus without schizophrenia had 63% increased plasma levels of CRP (P = 1 x 10^-4^). A hypothesized biological mechanism for this finding was the disruptive effect of CRP on the blood brain barrier, which might lead to increased permeability for pro-inflammatory cytokines and/or autoantibodies. Also Weiser et al. (Weiser, 2014) studied, in a post-hoc analysis, the effect of add-on aspirin in schizophrenia and whether any effects are moderated by plasma CRP levels, hypothesizing that higher levels (CRP>3850 ng/ml; >3.9mg/L) might reflect a higher level of inflammation, predicting a better response. Indeed, a subgroup of patients with relatively high levels of CRP was found to have significant improvements in positive symptoms of psychosis when treated with aspirin compared to patients with a low or intermediate CRP level.

Other support for an inflammatory component comes from genetic studies. A large pooled data-set of single nucleotide polymorphism (SNP)-based genome-wide association studies followed up the most significant association signals (Stefansson et al., 2009). One of the most remarkable findings was a significant association with several markers spanning the major histocompatibility complex (MHC) region on chromosome 6p21.3-22.1, which has also been linked to immune disorder. Therefore, this genetic deviation in the MHC region is consistent with an immune component to schizophrenia risk.

Finally, immune dysregulation might be reflected by abnormal levels of cytokines and the presence of auto-antibodies in serum and cerebrospinal fluid (CSF) (Bechter et al., 2010). This mounting body of evidence suggests that anti-inflammatory drugs can be viewed as potential candidates for new augmentation therapies; although at this stage it is unknown if increased pro-inflammatory status is characteristic for all patients with schizophrenia or just for a specific subset of them. It has long been recognized that part of the patients currently diagnosed as having schizophrenia has in fact a secondary psychosis, caused by inflammatory causes including auto-immune encephalitis and viral encephalitis. A recent study by Steiner et al. (Steiner et al., 2013) showed that 9.9% of patients with schizophrenia could be determined to suffer from auto-immune N -methyl-D-aspartate glutamate receptor (NMDA-R) encephalitis, against 0.4% in healthy controls. An older study collecting liquor found that 28.6% of patients with recent onset schizophrenia had a diagnostic (fourfold) change in the antibody titer in the paired serum CSF samples for different viruses including herpes simplex, varicella zoster, measles and cytomegalovirus (Srikanth et al., 1994). For these specific subgroups, a very large effect of immune modulation is expected, as it may positively affect the primary cause of psychosis.

In a longitudinal nested case-control study by Laan et al. (Laan et al., 2009), the diagnosis of psychotic disorder was investigated in a large population using glucocorticosteroids, both systemic and inhaled, for a somatic disorder. It was shown that glucocorticosteroid use was associated with a significantly lower risk of psychosis in men, finding Odds ratios around 0.50, suggesting a protective effect. Decreasing risks were associated with increasing dose of glucocorticosteroids. To date no additional studies have been performed regarding these indicative findings concerning potent immunomodulators such as glucocorticosteroids. A recent meta-analysis (Sommer et al., 2014) did study the efficacy of various other anti-inflammatory agents, in addition to antipsychotic treatment, to reduce symptom severity in schizophrenia. Aspirin addition showed promising results. In a previous meta-analysis (Sommer et al., 2012) five randomized studies applying non-steroidal anti-inflammatory drugs (NSAIDs) to patients with schizophrenia have been published; four using the selective cycloxygenase-2 (COX-2) inhibitor celecoxib and one using aspirin. A significant beneficial influence of these drugs, with small to moderate effect sizes on positive (0.34), negative (0.26) and total (0.43) symptom severity was demonstrated. The data was heterogeneous, with duration of illness as a potential moderator. We therefore concluded that the effect of immune modulation may be largest in patients with recent onset schizophrenia, as the hypothesized pro-inflammatory status in the brain may be most pronounced during and shortly after disease onset (Sommer et al., 2012). The significant effect of NSAIDs on total symptom severity indicates a positive influence of treatment involving immune modulation. However, prostaglandins and their receptors are found throughout the body and are involved in many regulatory processes, including blood clotting, vasoconstriction/dilatation, pain perception and thermal regulation. It remains unclear if the amelioration of symptoms is due to the associated immunosuppressive actions or to induction or blockade of other functions of prostaglandins. Biological immune modulators on the other hand, such as infliximab, etanercept and rituximab, are potent immunosuppressive agents, although they do not pass the blood-brain barrier (Tweedie et al., 2007). Another disadvantage of these biologicals is that they specifically target only one part of the immune system.

Therefore, it will be important to investigate the efficacy of administering a broad-acting, potent immune suppressive agent early in the course of the disease as this may prevent neuronal damage caused by low-grade inflammatory processes in the brain. In imaging studies on schizophrenia progressive gray matter loss is seen in most patients after the first psychotic episode. In several studies this gray matter loss in the first five years after diagnosis is associated with poor cognitive functioning and bad outcome (Lieberman et al., 2005, Cahn et al., 2006, van Haren et al., 2007). If immune modulation can avert loss of gray matter, this would be expected to be most effective to prevent further brain volume loss in these first five years of illness. It is expected that both symptom severity and long-term outcome will be improved. Cognition may also be a good predictor of long-term functional outcome (Schmidt et al., 2011).

Corticosteroids are potent immune modulating agents that target many different aspects of both the innate and the adaptive immune responses. Although the indications of immune involvement in schizophrenia are accumulating, it is still not possible to reliably specify exactly which components of the immune system are involved (Chan et al., 2011).This leads to uncertainty in the choice of targets. Corticosteroids are effective in various chronic inflammatory and auto-immune disorders (Lowenberg et al., 2008). Studies in patients with multiple sclerosis showed a positive effect of corticosteroid treatment on cognition (Zephir et al., 2005, Zephir et al., 2008), indicating that corticosteroids can prevent cognitive deterioration in diseases with an inflammatory component. So far, no robust data exists on the effectiveness of corticosteroids in schizophrenia. Between 1950 and 1960, several small studies assessed the effect of corticosteroids in patients with schizophrenia. However diagnostic standards have changed substantially over time. Also in these studies only short term administration of corticosteroids were studied. Furthermore, these studies focused on chronically ill patients, while recent data suggest that the beneficial effects of immune suppression may be largest in the early stage of the disease (Sommer et al., 2012).

Due to stronger mineralocorticoid potencies, several corticosteroids such as cortisone and cortisol are less preferable to include as immune-modulating agent in this proof of concept study. Mineralocorticoids mainly have an effect on electrolyte and water homeostasis while glucocorticoids are associated with anti-inflammatory, immunosuppressive activity. Prednisolone has only slight mineral-corticosteroid potency. It interferes with almost all primary and secondary immune cells, including monocytes, microglia cells, T-cells and granulocytes (Spies et al., 2010). Furthermore prednisolone can easily pass the blood-brain-barrier, which is a prerequisite to induce immune modulation in the brain. Finally, there is ample clinical experience with prednisolone and its side effect and safety profiles are well known (Ravindran et al., 2009). Neuropsychiatric side effects have been reported, in a dose dependent fashion. In a recent meta-analysis, hazard ratios for all neuropsychiatric side effects, except panic disorder, did not exceed 1.3 for dosages up to 40mg prednisolone (Fardet et al., 2012).

Therefore, we propose to investigate the effects of administering the corticosteroid prednisolone versus placebo in addition to standard antipsychotic medication in patients with early stage schizophrenia or related disorders, hypothesizing that a decrease in the overall low-grade cerebral inflammation due to prednisolone treatment will be expressed as a decrease in overall symptom severity. Hypothesizing that higher levels of CRP might reflect a relatively high inflammation state, we will select patients based on an elevated CRP level and therefore expect to target a subgroup of psychosis patients more likely to respond well to immune modulation, Secondly, addition of prednisolone is hypothesised to slow down cognitive deterioration in recent-onset psychosis patients. Finally, we aim to determine whether indirect immunological parameters of the hypothesised low grade inflammation status in schizophrenia are shifted due to the addition of prednisolone. A broad panel of blood markers will therefore be studied on the basis of which a prediction model for symptom improvement due to prednisolone treatment will be developed.

# OBJECTIVES

**Primary Objective**: The primary objective of this trial is to investigate whether prednisolone improves overall symptom severity as compared to placebo when given for 3 days in a dosage of 40 mg and subsequently tapered during 6 weeks in addition to antipsychotic medication to patients with early-stage psychotic disorder. We expect to find an improvement of symptoms as measured by the Positive And Negative Syndrome Scale (PANSS) compared to baseline, over the course of 6 weeks (Kay et al., 1987).

**Secondary Objectives**: Secondary objectives concern PANSS-scores after 6 and 12 months of follow up, improvement in cognitive functioning as measured by the Brief Assessment of Cognition in Schizophrenia (BACS). In addition, the positive and negative symptoms as well as general psychopathology (through PANSS subscales) are compared between treatment groups, next to general functioning using the GAF (Global Assessment of Functioning) (Karterud, 1998). Severity of depression will be assessed using the Calgary Depression Scale for Schizophrenia (CDSS) (Addington et al., 1990). Also the characteristics of patients (potentially) benefiting more from immunemodulation will be investigated through the assessment of a broad panel of immune parameters in blood. Finally, safety data will be evaluated by comparing incidences (number and % of subjects with at least one occurrence) of key SAEs and SUSARs between both groups, e.g. hospitalisations.

# STUDY DESIGN

In the current study, we aim to investigate the effect of additional treatment with prednisolone on symptomatic improvement, global functioning, cognition and on immunological parameters in patients with early-stage psychotic disorder, applying a randomized double-blind placebo-controlled add-on design. A placebo-controlled design was chosen in order to differentiate between clinical effects of prednisolone and effects associated with experimental treatment, such as induced expectations of participants. Randomization is applied to minimize bias. Prednisolone or placebo is provided *next* to existing antipsychotic medication as we do not intend to replace existing treatment, this study being a Proof of Concept trial. It would carry considerable risks for patients to taper down existent antipsychotic medication and randomize patients to either placebo or a type of therapy for which the efficacy still has to be proven, even for a short period of time. Finally, by applying the placebo arm and urging treating physicians to keep the antipsychotic dose as stable as possible during the 6 week period, the effect of prednisolone addition can be elegantly measured. Several aspects of the study design are implemented to ensure patient safety. For instance patients will be assessed weekly to provide accurate monitoring, and recruitment will predominantly take place among in-patients to ensure close follow-up in the first weeks of the study. By keeping the treatment phase relatively short and given dosage will be kept in the low to medium range (referring to the guideline for inflammatory bowel disease) we intend to optimize safety measures.

90 patients with schizophrenia, schizoaffective or schizophreniform disorder, or psychotic disorder NOS (not otherwise specified) will be included, with an age of 18-70 years and a time interval between the onset of psychosis and study entry not exceeding five years. A minimum plasma level of CRP, a marker of general low grade inflammation state, is required. Patients with a CRP > 3.9 mg/L will be included, hereby referring to the study of Weiser et al. (Weiser, 2014) . All 90 in- and outpatients will be randomized 1:1 to either prednisolone or placebo daily for 6 weeks. Prednisolone will be initiated at 40mg/day for 3 days and the 4 remaining days of the first week 30mg/dag will be used. After this first week, the medication will be tapered down 5mg/day per week. That is, the second week, patients use 25 mg/day, the third week 20 mg/day etc. Patients will continue their antipsychotic medication and (if applicable) other psychotropic drugs throughout the treatment period of 6 weeks. Doses are preferably kept stable if the clinical condition does not indicate a dose/ drug change, but dose adjustments are allowed as described in section 6.2 (use of co-intervention). At baseline psychiatric symptoms will be assessed using the PANSS and GAF questionnaires. Also, the Calgary Depression Rating Scale for Schizophrenia (CDSS; (Addington et al., 1990) and a neurocognitive assessment will be performed. These tests will be repeated during the treatment period and at the 6 week and 6-month follow-up evaluations. The measurement of various immunological biomarkers will be performed, to analyse changes over time as well as identify immune profiles that may be more susceptible for immune-modulating treatment. Blood samples will be drawn at baseline and after 3 and 6 weeks treatment, and after 6 months follow up to perform the measurement of immune parameters. During the treatment period, patients will be seen at weekly intervals to assess symptom severity, depressive mood and suicidal ideation, global functioning and side effects.

For this proof of concept study, a treatment period of 6 weeks is appropriate. We hypothesize that if prednisolone carries the expected effect, this will be apparent within this period of time. The study does not intend to assess the therapeutic potential of prednisolone as a possible augmentation therapy for these patients. Rather, the results may strengthen the case for immune-modulatory treatment and encourage further research in this direction.

# STUDY POPULATION

## 5.1 Population

90 patients with an age of 18-70 years will be included, diagnosed with either schizophrenia, schizoaffective or schizophreniform disorder (DSM-IV 295.*) or psychotic disorder NOS (not otherwise specified) (298.9). The onset of psychosis should be no more than five years ago. Antipsychotic treatment will be kept as stable as possible, but limited dose changes are allowed (for specifics, please refer to section 6.2: use of co-intervention). Patients will predominantly be recruited from the participating hospitals’ in-patient clinic, but also from outpatient clinics. In addition, the patient and family associations will be involved in notifying patients with regards to the possibility of participating in this study, through their media. With 3 years of recruitment, the inclusion of 90 patients through the participating centres and patient/family associations should be feasible.

## 5.2 Inclusion criteria

In order to be eligible to participate in this study, a subject must meet all of the following criteria at visit 1:

1. A DSM-IV-R diagnosis of: 295.x (schizophrenia, schizophreniform disorder, or

schizoaffective disorder) or 298.9 (psychosis NOS)

1. Onset of psychosis no longer than 5 years ago
2. Minimum total PANSS score of 60 Age 18 -70 years.
3. Patients are treated with antipsychotic medication
4. Plasma level of CRP is > 3.9 mg/L at screening (through ‘high sensitivity’ measurement)
5. Written informed consent is obtained
6. Female patients of childbearing potential need to utilize a proper method of contraception

(the pill, vaginal ring, hormonal patch, intrauterine device, cervical cap, condom,

contraceptive injection, diaphragm) in case of sexual intercourse during the study.

## 5.3 Exclusion criteria

A potential subject who meets any of the following criteria will be excluded from participation in this study in case of:

1. Presence of any of the contra-indications of prednisolone as reported in the SPC. These include hypersensitivity to any ingredients in the formulation, systemic infections unless specific anti-infective therapy is employed, patients with ocular herpes simplex due to the possibility of perforation, recent vaccination with live or weakened virus or bacteria. Also the following special warnings in the SPC will represent exclusion criteria: Existing or previous history of severe affective disorders in themselves or in their first degree relatives, including depressive or bipolar disorders or previous steroid psychosis, glaucoma or family history of glaucoma, hypertension or heart failure, liver impairment and/ or failure, epilepsy, osteoporosis, peptic ulceration, previous steroid myopathy, renal insufficiency, history of tuberculosis or x-ray changes characteristic of tuberculosis, recent myocardial infarction, chickenpox, measles.
2. Presence of diabetes mellitus or random (non-fasting) glucose levels exceeding 11 mmol/L at screening, or family history of diabetes.
3. Body Mass Index (BMI) of >27.5
4. Current or chronic use of systemic glucocorticosteroids (temporary use is permitted, if stopped before start of treatment trial)
5. Chronic use of non-steroidal anti-inflammatory drugs, defined as daily use during more than 2 months. Intermittent use is permitted, if stopped at least 1 month before start of treatment trial.
6. Pregnancy or breast-feeding. A urine pregnancy test will be performed at screening and then after 6 weeks of treatment and the event of treatment discontinuation.
7. Concurrent use of certain types of medication:
8. liver enzyme inducing medication such as carbamazepine, riphampicine, primidone, barbiturates and phenytoine
9. HAART (both HIV protease inhibitors and (non)-nucleoside reverse transcriptase inhibitors), especially efavirenz, ritonavir and lopinavir.
10. telaprevir and boceprevir in treatment of Hepatitis C

## 5.4 Sample size calculation

A two group t-test with a 0.050 two-sided significance level will have 80% power to detect an effect size of 0.610 when the sample sizes in the two groups are 44 and 44, respectively (a total sample size of 88) (text generated by nQuery Advisor). Subsequently the sample size can be reduced by the data with an Ancova instead of analyzing a t-test (Borm et al., 2007) (sample size reduction factor)). The level of reduction depends on the correlation (r) between baseline and follow up measurements: N-ancova = ((1-r^2^) * N-t-test) + 1.From a previous study (Optimise study(ClinicalTrial.gov identifier: NCT01248195)) where a PANSS was performed at baseline and after 6 weeks, r = 0.48 was found. Therefore, N-Ancova = ((1 – 0.48^2^ ) * 88) + 1 = 68.7248. Moreover in this study we will use a mixed model, however since we do not estimate a linear change in this analysis, the increase in efficiency compared to the Ancova and therefore the reduction in sample size will not be immense, this calculation will suffice.

Lastly we expect a considerable number of drop-out. Assuming a drop-out rate of 30 % the definitive sample size is calculated at 68.7248 * 1.3 = 89.34224, which makes a total of N = 90 patients.

(This power calculation was performed by Dr. C. van Baal, statistician at the University Medical Centre Utrect, Julius Center).

# 6. TREATMENT OF SUBJECTS

## 6.1 Investigational product/treatment

The main investigational product used in this trial is prednisolone, which is approved for systemic treatment for rheumatologic, pulmonal, gastrointestinal, endocrinologic, hematological, oncological, neurological, dermatological and opthalmological diseases. In addition, it is used topically in dermatology and rheumatology. It is also applied as an immunosuppressant in organ transplantation. In the current study, prednisolone will be initiated during the first week at 40mg/day for 3 days and 30mg/day for 4 days, followed by a decrease of 5mg/day per week during the remaining 5 weeks; in the second week, patients will use 25 mg/day, in the third week 20 mg/day is used etc. In the last week the patients will only take prednisolone on day 1-3 and day 5 and 7; a tapering scheme in line with the treatment guidelines for Inflammatory Bowel Diseases (Irving et al., 2007), <http://legemiddelhandboka.no/Terapi/17377?expand=1>. Also we have consulted physicians of various specialities (gastro-enterology, immunology, rheumatology) who use prednisolone in daily practice for approval of the schedule. Additionally, a placebo product is used consisting of an inactive substance (filler) which is identical to the prednisolone tablet

## 6.2 Use of co-intervention (if applicable)

Treatment with antipsychotic medication and (if applicable) other psychiatric medication will be continued as prescribed by the treating physician and will preferably be kept stable (i.e. same agents and dose) during the 6-week treatment period. We will allow the use of all types of antipsychotic drugs within the dose limits approved for registration. However, benzodiazepine use will be allowed to a maximum mean daily dose equivalent to 50 mg oxazepam. Changes in dosage of antipsychotic medication or benzodiazepines of at least 25% (relative to the screening visit), or change of antipsychotic drug, will be regarded as a secondary outcome measure. (To prevent osteoporosis, calcium suppletion (calciumcarbonate 500mg, daily) and vitamin D suppletion (colecalciferol 400ie, daily) will be provided to all patients, referring to the treatment recommendations for glucocorticoid treatment) <http://legemiddelhandboka.no/Legemidler/43999>

.

## 6.3 Escape medication (if applicable)

Pantoprazole, to treat possible side effects of the investigational product (ulcerative oesophagitis have been described as a rare adverse effect of glucorticocoids), is allowed to a maximum daily dose of 20 mg. Pantoprazole will be actively provided to patients with a history of ventricular/duodenal ulcer.***7.* INVESTIGATIONAL** **PRODUCT**

## 7.1 Name and description of investigational product(s)

Prednisolone belongs to the group of corticosteroids, synthetic agents which are derivatives of cortisol, a steroid hormone produced by the adrenal cortex. Prednisolone has predominantly glucocorticoid and minimal mineralcorticoid properties. It is mostly used for oral administration. Preferably capsules are ingested before or during meals with water or milk. During symptoms of gastrointestinal reflux, ingestion after meals is recommended. If titrated to higher dosages prednisolone preferably needs to be administered twice daily or more frequent.

The investigational product is approved by the Norwegian Medicines Agency (NoMA) (https://www.legemiddelsok.no/_layouts/15/Preparatomtaler/Spc/0000-06689.pdf) for various disorders with the aim of anti-inflammatory and immune suppression effects. See also (https://www.medicines.org.uk/emc/medicine/24130). Furthermore, a placebo product is used consisting of an inactive substance (filler) which is identical (e.g. sight, smell, weight) to the prednisolone product. The double blind medication is manufactured by Kragerø Tablettproduksjon AS and will be provided in blisters which are labeled in accordance with the Clinical Trial Directive requirements.

## 7.2 Summary of findings from non-clinical studies

Corticosteroids such as prednisolone are drugs that have been available for a substantial number of years and consequently a wide range of toxicological studies have been undertaken in both man and animals. It has been demonstrated that prednisolone may cause a cleft palate in rats and mice. In high dosages, descendants of mice showed hypospadia, and descendants of rats demonstrated constriction of the ductus arteriosus. In animal studies, intrauterine growth retardation has been found.

## 7.3 Summary of findings from clinical studies

The introduction of glucocorticoids in the 1950s was a revolution in the treatment of a large variety of inflammatory diseases. To this day, corticosteroids represent a frequently used and effective class of anti-inflammatory drugs, in numerous neoplastic, immunological and neoplastic diseases (Fardet et al., 2007). Glucocorticoids are estimated to be used long term by 0.5-1.0% of the general population and up to 2.5% of older adults (Curtis et al., 2006).

## 7.4 Summary of known and potential risks and benefits

The well-established efficacy of prednisolone to lower the immune response is counterbalanced by a considerable amount of side-effects which have been well recognized over the years. There remains debate on the true incidence of side effects since it is difficult to separate the side effects from the outcome of the underlying disease. Some indication for a dose-response relationship was found with regards to side effects of glucocorticoids in general (McDonough et al., 2008), although this was not demonstrated in other studies.

Due to the immunosuppressive capacities of corticosteroids, decreased resistance to infections can occur; Fardet (Fardet et al., 2007) reported in his review a relative risk of 1.6 for bacterial sepsis. Reactivation of latent tuberculosis is a much debated adverse effect, incidence particularly increasing in dosages >15mg during long-term administration. Osteoporosis is a well-established, time and dose dependent side effect of chronic glucocorticoid use (Da Silva et al., 2006). Another common adverse effect of corticosteroids is symptoms of Cushing’s syndrome. Most recognizable is a cushingoid habitus where adipose tissue accumulates in facial, dorsocervical and abdominal regions, whereas subcutaneous fat thickness is reduced in the limbs. These symptoms are usually reversible when they emerged during prednisone equivalent daily dosages of <10mg/day (Fardet et al., 2007). No such adverse effects have been observed in a corresponding study already ongoing in the Netherlands, which have included 20 patients thus far (Iris Sommer, Principal Investigator, the **Prednisolone addition for patients with recent-onset psychotic disorder** study (Eudra CT-number 2014-000520-14), Personal communication). Risk of cardiovascular events is increased in corticosteroid use, with a threefold higher risk for patients with glucocorticoid induced lipodystrophy. Hypertension is a well-known adverse effect in steroid use, often already seen after early initiation of corticosteroids. Although its frequency is variable and dependent on dosage and duration, an odds ratio of 1.3-2.6 was reported by Fardet (Fardet et al., 2007). In a study by Sato (Sato et al., 1995) in patients over 65 years of age, corticosteroid-induced hypertension was only seen in dosages >20mg/day of prednisone equivalent, with low serum calcium levels as a risk factor. Increase of glucose serum levels is also common in glucocorticoid use, causing insulin resistance. Corticosteroids may induce a worsening of glycaemic control in patients with known diabetes. Although glucocorticoid related hyperglycemia is dose dependent, it is also reported in low doses (Da Silva et al., 2006). Davenport (Davenport et al., 2011) studied short-term administration of high dosed corticosteroid (600mg hydrocortisone) given prior to administration of radiologic contrast medium, causing development of brief and reversible hyperglycemia. However a study by den Uyl et al. (den Uyl et al., 2012) showed that short term treatment with prednisone at 60 mg or 30mg per day improved disease activity without deterioration of glucose tolerance in patients with active RA. Also Yates et al. (Yates et al., 2014) found that divided daily dosing of prednisolone reduces glycaemic variability and hyperglycaemia in patients after kidney transplantation.

In corticosteroid use also neuropsychiatric adverse events and behavioral disturbances have been reported (Hoes et al., 2009, Fardet et al., 2012), ranging from mild irritability to suicidal ideation and psychosis. Risk for neuropsychiatric outcomes were higher for patients with a previous history of neuropsychiatric disorders and those treated with high dosages of glucocorticoids (Fardet et al., 2012).

Meanwhile a study by Laan et al. (Laan et al., 2009) showed that the use of glucocorticosteroids, both systemic and inhaled, for a somatic disorder significantly lowered risk of psychosis in men, with odds ratios around 0.50, suggesting a protective effect of glucocorticosteriod treatment. Decreasing risks were found with increasing dose of glucocorticosteroids. In most of the above mentioned studies higher dosed and/or chronic use of corticosteroids were studied. In the current study the maximum dosage of 40 mg is only given during 3 days, hence while we are cautious of adverse effects, such quantities or risks as mentioned above are not expected.

##

## 7.5 Description and justification of route of administration and dosage

Route of administration is orally through the use of tablets, containing either prednisolone or an inactive substance (placebo). Preferred time of ingestion is before or during meals. If titrated to higher dosages prednisolone preferably needs to be administered twice daily. In patients who have received more than physiological doses of systemic corticosteroids (approximately 7.5mg prednisolone) for greater than 3 weeks, withdrawal should not be abrupt. Therefore, this study will use an elaborate titration scheme.

## 7.6 Dosages, dosage modifications and method of administration

Subjects will ingest the products in the form of identical tablets. In the current study, the following titration schedule will be employed in line with the treatment guidelines for Inflammatory Bowel Diseases (Irving et al., 2007), <http://legemiddelhandboka.no/Terapi/17377?expand=1> which should minimize the occurrence of any side effects and withdrawal symptoms. Also we have consulted physicians of various specialities (gastro-enterology, immunology, rheumatology) who use prednisolone in daily practice for approval of the schedule.

1. *week 1:*

day 1-3: 40 mg/day, divided over two intakes

day 4-7: 30 mg/day, divided over two intakes

1. *week 2:*

25 mg/day, divided over two intakes

1. *week 3:*

20 mg/day, divided over two intakes

1. *week 4:*

15 mg/day, divided over two intakes

1. *week 5:*

10 mg/day, intake once daily

1. *week 6:*

day 1-3: 5 mg/day, intake once daily

day 4-7: on day 5 and 7, 5 mg/day, intake once daily, on day 4 and 6 no tablets.

After week 6, prednisolone will be discontinued. These dosages are within the registered therapeutical dose range for prednisolone, which varies from 10 to 120mg/daily depending on disease severity.

7.6.1 Choice of dosing of prednisolone

There is no established dose interval for prednisolone in schizophrenia and related disorders as the study represents a new line of research in the pharmacological treatment of these disorders. In the studies on methylprednisolone in multiple sclerosis considerably higher doses were used than what is planned in the current study in schizophrenia (Zephir et al., 2005, Zephir et al., 2008). The choice of dose interval in the present study is a result of a close risk-benefit consideration. Higher doses are associated with higher risks of side effects presumably including also mental adverse effects on the one hand. On the other hand the dose needs to be sufficient to provide an anti-neuroinflammatory effect. The elevated inflammatory state associated with psychosis is generally found to be of low-grade, see also section 2. Some of the agents that have yielded promising yet equivocal results when added to antipsychotics are of lower anti-inflammatory potency compared to prednisolone (Sommer et al., 2014), indicating that a dosage in the middle of the dosing interval of prednisolone should be sufficient to provide an anti-inflammatory effect in schizophrenia. In summary the dosing regimen is considered sufficient to dampen low-grade neuro-inflammation, but at the same time keeping the risk of side effects at a low level.

## 7.7 Preparation and labelling of Investigational Medicinal Product

Study medication will be provided by Kragerø tablettproduksjon AS. Tablets will be packed in week-kits, labeled in line with Annex 13 of the Clinical Trial Directive.

## 7.8 Drug accountability

Study medication will be delivered by the pharmacy of Kragerø tablettproduksjon AS to the researchers at the participating centers. Medication will be stored either at the local pharmacy or in a locked medicine cabinet in a locked room at the Psychiatry Department with limited access and monitored (room) temperature. The temperature log will be checked weekly. Only two permanent members of the study team will have possession of the keys. Drug receipt, dispense, return and destruction will be recorded in medication log files, which will be checked by the study monitor. The patients will be provided with sufficient study medication until the next dispensing visit, including spare medication to overcome a potential delay until the next visit. All unused medication will be returned by the patient to the investigator at the next visit, and new medication will be provided in accordance with the protocol. Accountability and subject compliance will be assessed by maintaining adequate drug dispensing and return records, as described in detail in the Standard Operating Procedure (SOP) Medication Management.

The inventory will be available for inspection by the monitor. All supplies, including empty containers, must be returned at the end of the study. Drug accountability logs will be archived together with the other study documentation. Medication Management is described in more detail in the separate SOP dedicated to these procedures.

# 8. METHODS

## 8.1 Study parameters/endpoints

### 8.1.1 Main study parameter/endpoint

Our main study parameter is overall symptom severity as measured with the Positive and Negative Syndrome Scale (PANSS) total score (Kay et al., 1987). We will compare the effect of prednisolone versus placebo, both given in addition to antipsychotic medication, with regards to change in overall symptom severity, measured after 6 weeks of treatment compared to baseline.

### 8.1.2 Secondary study parameters/endpoints

Secondary study parameters include PANSS total scores 6 months after start of the treatment, neurocognitive functioning as measured with the Brief Assessment of Cognition in Schizophrenia (BACS) and symptom severity as measured with the PANSS subscales: the positive scale, negative scale and general psychopathology scale. Furthermore, general functioning will be evaluated using the split- GAF (Karterud, 1998). These parameters will be compared between patients treated with prednisolone versus placebo. Various serum and peripheral blood mononuclear cells will be collected from all patients at baseline, as well as after 3 and 6 weeks of treatment and after 6 months of follow up. The specific lab assessments are described in section 7.3. Furthermore, severity of depression will be assessed and compared between groups using the CDSS. The need to adjust current antipsychotic medication with 25% or more of the dose in Defined Daily Doses (DDD) is compared between groups. Finally, safety data will be assessed by comparing incidences (number and % of subjects with at least one occurrence) of key SAEs and SUSARs between both groups, e.g. hospitalisations.

## 8.2 Randomisation, blinding and treatment allocation

### 8.2.1 Randomization

Randomization will be performed centrally under the supervision of Kompetansesenteret for klinisk forskning, Haukeland Unversity Hospital. Stratification will be applied for sex. Blockrandomisation will be performed for sex and different centers. Trial treatment randomization codes will not be available to the study staff. The randomization lists will be sent in a sealed envelope to Kragerø tablettproduksjon AS for preparation of the study medication/ placebo. Also, Kragerø tablettproduksjon AS will make individual sealed envelopes containing the result of the randomization for all participants for use in an emergency unblinding situation, see 8.2.2. for further details.

### 8.2.2 Unblinding Procedure

Emergency unblinding may only occur on an individual basis for safety reasons if the information can help the treatment of an (S)AE. For SUSARs, unblinding is always required. For emergency unblinding, envelopes are available at the Psychiatric clinic, Division of Psychiatry, Haukeland University Hospital. An allocated person and a back-up person with access to the unblinding envelopes will be available 24/7. In the case of a medical emergency situation as described below, the sealed envelope can be opened and the treatment assignment of a participant will be unblinded. In case of an emergency unblinding, the reason and time of unblinding will be documented in the study files. The decision to unblind is at the discretion of the investigator.

Emergency unblinding is indicated in the following situations only:

1. unblinding is necessary for the subjects emergency treatment at the investigators discretion
2. unblinding is required by local laws or regulations (in case of SUSAR)

Opened envelopes will be archived in the Investigator Site File at the study site. A more detailed description of unblinding procedures can be found in the SOP Medication Management.

## 8.3 Study procedures

Study examinations scheduled in the course of the trial are listed in table 1 below. Patients will come to the study center for each visit. If preferred by the patient and if study procedures allow it, an experienced researcher can visit the patient at home.

**Table 1: Patient visits and examinations specified per visit**

|  |  | **Weeks** | **Informed consent, in-/ exclusion criteria, MINI, demographics** | **Medical history, current medical conditions, physical examination , BMI, ECG** | **Pregnancy testing** | **Use of concomitant medication** | **Dispense study medication** | **Randomization** | **Drugs/alcohol use** | **Side effects, compliance** | **PANSS, GAF** | **Suicidal behavior, CDSS, hospitalisation** | **BACS** | **Blood/ urine samples** |
| --- | --- | --- | --- | --- | --- | --- | --- | --- | --- | --- | --- | --- | --- | --- |
| **Visit 1** | **Screening** | **-12**  **to 0** | **X** | **X** | **X** | **X** |  |  | **X** |  | **X** |  |  | **X** |
| **Visit 2** | **Baseline** | **0** |  |  | **X** | **X** | **X** | **X** |  |  | **X** | **X** | **X** | **X** |
| **Visit 3** |  | **1** |  |  |  | **X** | **X** |  |  | **X** |  | **X** |  | ****** |
| **Visit 4** |  | **2** |  |  |  | **X** | **X** |  |  | **X** | **X** | **X** |  | ****** |
| **Visit 5** |  | **3** |  |  |  | **X** | **X** |  | **X** | **X** |  | **X** |  | **X** |
| **Visit 6** |  | **4** |  |  |  | **X** | **X** |  |  | **X** | **X** | **X** |  | ****** |
| **Visit 7** |  | **5** |  |  |  | **X** | **X** |  |  | **X** |  | **X** |  | ****** |
| **Visit 8** |  | **6** |  |  | **X** | **X** |  |  | **X** | **X** | **X** | **X** | **X** | **X** |
| **Visit 9** |  | **26** |  |  |  | **X** |  |  |  |  | **X** |  | **X** | **X** |
| **Visit 10** |  | **52** |  |  |  | **X** |  |  |  |  | **X** |  |  |  |
|  | **Early termination visit** | ***** |  |  | **X** | **X** |  |  | **X** | **X** | **X** | **X** | **X** | **X** |

*will be performed if a patient prematurely discontinues the study.

**fasting glucose only.

MINI =Mini International Neuropsychiatric Interview; BMI=Body Mass Index; ECG=electrocardiogram; PANSS=Positive And Negative Syndrome Scale; GAF=General Assessment of Functioning; CDSS=Calgary Depression Scale for Schizophrenia; BACS= Brief Assessment of Cognition in Schizophrenia.

**Screening visit (visit 1)**

After the informed consent procedure has been completed, in- and exclusion criteria will be checked to assess the patient’s eligibility for participation. The Mini International Neuropsychiatric Interview 5.0.0 (M.I.N.I. 5.0.0) will be administered to confirm the inclusion diagnosis (Sheehan et al., 1998). PANSS and GAF will be assessed. In addition, several demographical and clinical variables will be assessed, including date of birth, sex, educational level, prior psychiatric disorders, duration of untreated psychosis and use of drugs and alcohol. Furthermore, the use of concomitant medication, medical history and current medical conditions will be recorded. The maximum period between screening visit and baseline visit is three months. If the patient uses co-medication at the screening visit which is not allowed, it can be washed out during this period. A physical examination will be conducted, including BMI, blood pressure and ECG; abnormalities will be discussed with the patient’s treating physician. Signs of lipodystrophy will be observed. Also blood will be drawn to determine the plasma CRP-level (high sensitivity) and to rule out hyperglycaemia as well as signs of systemic infection as these are special warnings for the use of prednisolone. Additionally several blood parameters (blood differentiation, electrolytes, thyroid, liver and renal function) will be checked at screening to provide a screen for serious other diseases. These results will be evaluated before the first intake of study medication.

**Baseline visit (visit 2)**

At baseline, PANSS and GAF will be administered, in addition to the CDSS and the BACS (see below for details of these scales). If the time between screening and baseline is less than 2 weeks the PANSS will not be repeated at baseline. In addition, the presence of any exclusion criteria for the current study will be checked as well as suicidal ideation. The patient will be randomised and the study medication will be dispensed for the first time. Patients will be instructed on medication use, with regard to contraindicated co-medications (section 4.3). Blood will be drawn to assess immunological parameters. A study participation letter will be send to the treating psychiatrist, general practitioner and pharmacist regarding the patient’s participation.

**Treatment visits (visits 3-8)**

After the screening and baseline visits, patients will be assessed weekly for 6 weeks. During various visits, an experienced physician will interview them using the PANSS, GAF (every other week; visit 2-4-6-8) and CDSS questionnaires (weekly). At each visit, presence of suicidal ideation is carefully examined. Co-medication use will be noted as well as side effects and treatment compliance. Except for visit 8, study medication will be dispensed during each visit during the treatment period. Alcohol and drug use will be assessed during visits 5 and 8, in addition to the second and third blood draws intended for immunological assessments. Cognitive testing will take place at the end (6 weeks) of the treatment period. In case of drop-out, an extensive Early Termination visit will be performed to finalize participation. If the patient is not willing to complete all measures, priority will be given to the PANSS and BACS.

**Follow-up visits (visits 9 and 10)**

After 26 and 52 weeks (relative to the baseline visit), follow-up visits will take place, during which the PANSS and GAF are assessed. In additional, the current use of medication will be noted. At visit 9, blood will be drawn to assess immunological parameters and the BACS will be repeated once more.

**Drug administration**

In the active substance condition, participants will be provided with blisters prednisolone tablets; the number of tablets and the dosing depends on the visit number due to the tapering of prednisolone. In the placebo condition, participants are given identical looking blisters with placebo tablets consisting of an inactive substance. The blisters contain sufficient medication until the next visit. Instructions for use are provided simultaneously.

**Rating scales**

1. Positive and Negative Syndrome Scale (Kay et al., 1987): this is a 30-item rating scale designed to measure severity of psychopathology in adult patients with psychotic disorder. Five components have been reported: positive, negative, depression, agitation-excitement, and disorganisation.
2. General functioning will be assessed using the GAF questionnaire (Karterud, 1998).
3. Severity of depression will be assessed using the CDSS (Addington et al., 1990)

Proper conduct of the PANSS interview, the instrument used to measure the primary study outcome, will be taught using instructional videos and checked via the assessment of a test video. Each participant in this PANSS training has to pass the exam before he/she can perform PANSS ratings for this study. Suicidal ideation is not assessed using a questionnaire but rather during an unstructured interview, addressing the questions suggested by the M.I.N.I. plus 5.0.0 scale, performed weekly.

**Cognitive assessment**

Neurocognitive functioning will be assessed at the baseline visit and at the end (6 weeks) of treatment with the BACS (Keefe et al., 2004). The BACS is a newly developed instrument that assesses the aspects of cognition found to be most impaired and most strongly correlated with outcome in patients with schizophrenia. The BACS has an expected administration time of 35 minutes.

Verbal memory: list learning

Patients are presented with 15 words and then asked to recall as many as possible, which will be repeated five times.  Measure: number of words recalled per trial, in any order.

Working memory: digit sequencing task

Patients are presented with clusters of numbers of increasing length and are asked to tell them in order from lowest tot highest. Measures: number of correct responses.

Motor speed: Token motor task

Patients are given 100 tokens and are asked to place them in a container as quickly as possible. Measures: the number of tokens correctly placed into the container.

Verbal fluency: category instances

Patients are asked to name as many words in a certain category in 60 seconds (supermarket items, tools). Measures: *score is reflected in the number of unique and appropriate answers per category.*

Verbal fluency: controlled oral word association test

Patients are asked to generate as many words as possible that begin with a given letter. Measures: *score is reflected in the number of unique and appropriate answers per category.*

Attention and speed of information processing: symbol coding

*Timed paper-and-pencil test in which respondent uses a key to write digits that correspond to nonsense symbols. A sheet with a 9 item key is provided, pairing digit 1–9 with a unique symbol; below are rows of numbers with blank squares beneath. The subject pairs each number with its unique symbol. Measures: score is reflected in the number of correct number-symbol pairs completed in 90 seconds.*

Executive functions: Tower of London

Patients are shown two pictures simultaneously with 3 pegs uniquely arranged in each picture. Patients are asked to give the number of times the balls in one picture need to be moved in order to make the arrangements identical on both pictures. There are 20 trials in with variable difficulty. Measures: number of correct answers.

**Blood samples**

Blood samples will be drawn at screening, baseline, after 3 and 6 weeks of treatment and at follow up after 6 months. At screening, laboratory blood testing will be performed at the Laboratory facilities of Haukeland University Hospital/ St. Olavs Hospital/ Stavanger University Hospital to rule out serious liver, kidney or muscle disorders and diabetes, as well as to assess the CRP level. At baseline and after 3 and 6 weeks of treatment, regular laboratory assessments will be repeated to evaluate the patients’ safety. Measurements will include complete blood count, glucose, HbA1c, electrolytes (sodium, potassium) and kidney function (creatinin, urea). Two unblinded study team members will review the results in relation to generally accepted reference values, within a few working days after the results become available. The study physicians, treating the patients, will remain blinded at any time. In case blood values fall outside these reference ranges, an endocrinologist, involved as a consultant in the study, will be immediately asked for advice. If deemed necessary, the patient will be treated and/or discontinued from the study. This decision will be made on an individual basis.

One serum separator tube (10 mL), two or three sodium heparin tubes (9 mL) and 1 paxgene tube will be collected at baseline, 3 and 6 weeks and 6 months post-baseline (table 2) to investigate a panel of possible biomarkers associated with treatment response as noted in section 7.1.3, using different assays.

Serum and PBMCs will be prepared and stored using standard operating procedures. In short: Serum will be prepared by the Laboratory facilities of Haukeland University Hospital/ St. Olavs Hospital/ Stavanger University Hospital and aliquots will be stored at -80^0^C. PMBCs will be prepared, frozen and stored by layering the blood from the heparin tubes on a ficoll density gradient. After centrifugation PBMCs will be collected, aliquoted in cryovials and frozen at -80^0^C. Within one week the PBMCs samples will be transferred to liquid nitrogen. RNA Paxgene tubes are stored for two hours at room temperature and subsequently stored for 24 hours at -20^0^C and subsequently at -80^0^C in the central biobank. RNA purification will be performed with RNA extraction kits.
Each blood draw will consist of 3-42.5 mL divided over 1 or 6 tubes.

**Table 2: Blood samples specified per visit**

|  |  | **Week** | **Tube** | Complete Blood count, CRP, ASAT, ALAT, γ-GT, AF, LDH, creatinine, ureum, CK, glucose, HbA1C,Na, K | complete blood count, glucose, HbA1c, elektrolytes (Na, K) and kidney function (creat, ureum)  , CRP | Flow cytometry | Gene expression profiling ; microRNA expression profiling | Infectious disease profling | Multiplex immunoassay;  Selective reaction monitoring |
| --- | --- | --- | --- | --- | --- | --- | --- | --- | --- |
| **Visit 1** | **Screening** | **-12 to 0** | **LHT 3 ml** | **1x** |  |  |  |  |  |
| **Visit 2** | **Baseline** | **0** | **LHT 3 ml** |  | **1x** |  |  |  |  |
|  |  |  | **SHT 9 ml** |  |  | **3x** | | |  |
|  |  |  | **SST 10 ml** |  |  |  |  | **1x** | |
|  |  |  | **PT 2.5 ml** |  |  |  | **1x** |  |  |
| **Visit 5** |  | **3** | **LHT 3 ml** |  | **1x** |  |  |  |  |
|  |  |  | **SHT 9 ml** |  |  | **2x** | |  |  |
|  |  |  | **SST 10 ml** |  |  |  |  |  | **1x** |
|  |  |  | **PT 2.5 ml** |  |  |  | **1x** |  |  |
| **Visit 8** | **End of treatment** | **6** | **LHT 3 ml** |  | **1x** |  |  |  |  |
|  |  |  | **SHT 10 ml** |  |  | **3x** | |  |  |
|  |  |  | **SST 9 ml** |  |  |  | |  | **1x** |
|  |  |  | **PT 2.5 ml** |  |  |  | **1x** |  |  |
| **Visit 9** | **Follow-up** | **6** | **LHT 3 ml** |  | **1x** |  |  |  |  |
|  |  |  | **SHT 10 ml** |  |  | **2x** | |  |  |
|  |  |  | **SST 9 ml** |  |  |  |  |  | **1x** |
|  |  |  | **PT 2.5 ml** |  |  |  | **1x** |  |  |
|  |  |  |  |  |  |  |  |  |  |

LHT=lithium heparin tube; SHT=sodium heparin tube; SST=serum separator tube; PT = paxgene tube; ASAT=aspartate aminotransferase; ALAT=alanine aminotransferase; γ-GT=gamma-glutamyl transpeptidase; CK=creatine kinase; CRP=C-reactive protein;.

Peripheral biomarkers

It is generally accepted that schizophrenia is a heterogeneous disease caused by distinct underlying mechanisms. In this study we aim to target inflammatory processes that

may be involved in a subpopulation of patients with psychotic disorder (Chan et al., 2011) and response to antipsychotics (Schwarz et al., 2012) using prednisolone augmentation. To investigate immunological biomarkers that predicts treatment response to prednisolone therapy we will collect serum, peripheral blood mononuclear cells (PBMC) and RNA of all patients at baseline (Table 2). To further understand the effect of prednisolone on specific biomarkers correlated with response to the proposed add on therapy, blood samples will be taken at baseline, after 3 and 6 weeks of treatment and at follow up after 6 months (Table

2).

The following assays will be performed:

1. *Multiplex immunoassay.* Levels of a specified panel of low-abundant analytes (including key cytokines, chemokines, hormones, growth factors, acute phase reactants known to be associated with schizophrenia (Schwarz et al., 2010) will be measured in 750μL baseline serum from all patients using for example the Human DiscoveryMAP® 250+ v 1.0. The Human DiscoveryMAP includes pro-inflammatory markers that have previously been shown to be altered by prednisolone therapy including C-reactive protein, TNF-alpha and IL-6 (Schmidt et al., 2007). At 3 and 6 weeks of treatment and at follow up after 6 months post-baseline analytes that predict treatment response at baseline will also be measured using multiplex/ELISA’s ( the following analytes will be included: CRP, interferon-γ, interleukin (IL)-1RA, IL-1β, IL-4, IL-6, IL-8, IL-10, IL-12, IL-13, L-17, tumor necrosis factor-alpha (TNF-α), macrophage migration inhibitory factor, S-100B).
2. *Infectious disease profiling:* Infectious agents have been associated with schizophrenia because of the relation of the disease with perinatal infections, season of birth and the association with specific neurotropic pathogens (Arias et al., 2012). To investigate whether prednisolone responsiveness is related to specific past or present infections, we will measure serology (specific IgG and IgM) and the presence (PCR) of specific infectious agents, including herpes simplex virus type-1 and -2, cytomegalovirus, Chlamydophila pneumonia, Chlamydophila psittaci and Toxoplasma Gondii at baseline.
3. *Flow cytometry:* Alterations in the number and functional responses of distinct sub-populations of blood cells have been reported in schizophrenia (Drexhage et al., 2011). We will therefore analyse whether these alterations predict treatment response by prednisolone augmentation using flow cytometry. The analysis will yield comprehensive information about the number of specific subsets of leukocytes as well as levels of surface markers reflecting the cellular phenotype in relation to prednisolone treatment, glucose transporters, signalling pathways, apoptosis and oxidative stress before and during prednisolone treatment.
4. *Selective reaction monitoring (SRM):* For potential candidate biomarkers of drug response identified by proteome profiling (LC-MS^E^) we will apply a mass spectrometry based technique known as SRM. SRM relies on the ability of mass analyzers to select a specific analyte (protein peptide) to quantify its absolute expression level, compared to an appropriate stable isotope-labelled peptide standard, accurately, for up to 30 multiplexed analytes in a single sample run. SRM will be applied to the PBMC samples from all 90 patients treated with prednisolone collected at baseline, 3 and 6 weeks of treatment and at 6 months post-baseline to validate and monitor prednisolone response biomarkers during therapy.

*Gene expression and MicroRNA profiling:* The RNA expression level of 43 immune-related genes is altered in peripheral blood cells of patients with schizophrenia (Drexhage et al., 2010). Moreover, a recent study identified down regulated miR-146a expression in PBMCs of patients with schizophrenia (Gardiner et al., 2012). To determine whether RNA expression of one or more of these genes and miRNA-146a expression predicts the treatment response to prednisolone, RNA expression of the set of 43 immune-related genes and miRNA-146a will be measured in total blood and monocytes isolated from PBMCs at baseline using Q-PCR. Expression levels of genes that predict treatment response will also be measured at baseline, 3 and 6 weeks of treatment and at 6 months post-baseline.

## 8.4 Withdrawal of individual subjects

Subjects can leave the study at any time for any reason if they wish to do so without any consequences. The investigator can decide to withdraw a subject from the study for urgent medical reasons.

Reasons to terminate a patient’s participation include but are not limited to:

1. The patient withdraws her/his consent
2. Intolerance to the study drug
3. Start of the use of non-steroidal anti-inflammatory drugs (NSAIDs), HAART, telaprevir, boceprevir, riphamicine, primidone, barbiturates and phenytoine
4. Administration of a live vaccine is needed

## 8.5 Replacement of individual subjects after withdrawal

In case a subject discontinues before the end of the study, no replacement is needed due to the methods that were selected for our statistical plan.

## 8.6 Follow-up of subjects withdrawn from treatment

In case of withdrawal due to adverse events the subject will be followed-up until the adverse events have abated or have reached a stable situation.

## 8.7 Premature termination of the study

The study can be terminated prematurely in case of a change in the risk profile of prednisolone, making it necessary to re-assess the use of the investigational product.

In case the entire study is discontinued, patients will be followed-up and/or treated as usual in normal daily practice, depending on the severity of the changes in risk profile and the extent to which this increase in risk is applicable for individual patients.

# SAFETY REPORTING

## 9.1 Section 10 WMO event

This study will be performed according to the Declaration of Helsinki (64^th^ WMA general assembly; Fortaleza, Brazil, October 2013) and the International Conference on Harmonisation – Good Clinical Practice (ICH-GCP). The definitions of adverse events and serious adverse events described in these guidelines will be used for the present study.

In accordance to section 10, subsection 1, of the WMO, the investigator will inform the subjects and the reviewing accredited METC if anything occurs, on the basis of which it appears that the disadvantages of participation may be significantly greater than was foreseen in the research proposal. The study will be suspended pending further review by the accredited METC, except insofar as suspension would jeopardise the subjects’ health. The investigator will take care that all subjects are kept informed.

## 9.2 AEs, SAEs and SUSARs

### Adverse events (AEs)

Adverse events are defined as any undesirable experience occurring to a subject during the study, whether or not considered related to the investigational product. All adverse events reported spontaneously by the subject or observed by the investiga­tor or his staff will be recorded.

### Serious adverse events (SAEs)

A serious adverse event is any untoward medical occurrence or effect that at any dose:

1. results in death;
2. is life threatening (at the time of the event);
3. requires hospitalisation or prolongation of existing inpatients’ hospitalisation;
4. results in persistent or significant disability or incapacity;
5. is a congenital anomaly or birth defect
6. any other important medical event that may not result in death, be life threatening, or require hospitalization, may be considered a serious adverse experience when, based upon appropriate medical judgement, the event may jeopardize the subject or may require an intervention to prevent one of the outcomes listed above.

SAEs that result in death or are life threatening should be reported expedited. The expedited reporting will occur not later than 7 days after the responsible investigator has first knowledge of the adverse reaction. In case additional information is required, this will be provided through an update report within the next 8 days (within 15 days in total).

### Suspected unexpected serious adverse reactions (SUSARs)

Adverse reactions are all untoward and unintended responses to an investigational product related to any dose administered.

Unexpected adverse reactions are SUSARs if the following three conditions are met:

1. the criteria for an SAE are met (see section 8.1.2);
2. it is plausible that the event is caused by the study medication;
3. the adverse reaction must be unexpected, that is, the nature and severity of the adverse reaction are not in agreement with the product information as recorded in the Summary of Product Characteristics (SPC) for prednisolone.

In case a SUSAR occurs, the patient has to be unblinded. If possible, this will be done in such a way that the study team members involved in the analyses and interpretation of the results remain blinded.

**Reporting procedures to the Norwegian Medicines Agency (NoMA)**

All suspected side effects that are mortal or life-threatening, and unexpected, shall be reported to the NoMA immediately and within 7 days at the latest after the side effect has been brought to the attention of the sponsor. Suspected adverse events that are serious and unexpected shall be reported within 15 days. Serious adverse events shall be reported collectively in the annual report/ final report. It is only SUSARs that are reported as separate reports.

## 9.3 Annual safety report

The sponsor will submit, once a year throughout the clinical trial, a safety report to the accredited ERB and competent authority. This safety report consists of:

1. a list of all suspected (unexpected or expected) serious adverse reactions, along with an aggregated summary table of all reported serious adverse reactions, ordered by organ system, per study;
2. a report concerning the safety of the subjects, consisting of a complete safety analysis and an evaluation of the balance between the efficacy and the harmfulness of the medicine under investigation.

## 9.4 Follow-up of adverse events

All AEs will be followed until they have abated, or until a stable situation has been reached. Depending on the event, follow up may require additional tests or medical procedures as indicated, and/or referral to the general physician or a medical specialist.

SAEs need to be reported until the end of the study. However, should the investigator become aware of an SAE or SUSAR that occurs within 30 days after stopping the study treatment, the event must be reported in accordance with procedures specified above.

## 9.5 Data Safety Monitoring Board (DSMB)

The safety of the study will be judged by an independent committee of experts (DSMB) established for this and a corresponding study already ongoing in the Netherlands (Eudra CT-number 2014-000520-14) on regular basis, at a frequency of at least once a year. The members of this board will have access to all safety and progress information (e.g. inclusion and drop-out rates) in the study. If deemed necessary, the DSMB members may review the unblinded study data. The DSMB will meet each time after 10 additional patients are recruited, but meetings may be more frequent depending on trial events causing safety concerns, the enrolment rate (much slower or faster than anticipated) or when the DSMB deems this to be appropriate for other reasons. The DSMB may suggest changes to the protocol or provide an altered judgement of feasibility if information from the annual safety report or new information about the study medication becomes available. No interim analyses are planned.

# 10. STATISTICAL ANALYSIS

A two-sided 0.05 level of significance will be used to declare treatment arms significantly different. Intention-To-Treat analyses will be conducted.

Descriptive statistics of continuous outcomes will be presented by treatment arm and include sample size, mean, median, standard deviation, minimum and maximum.

For categorical outcomes (diagnoses, handedness and sex), the number and percentage of subjects in each category will be presented by treatment arm. All statistical analyses will be performed using SPSS for Windows (version 20) or other widely accepted statistical or graphical software.

##

## 10.1 Primary study parameter(s)

The primary analysis will include the PANSS scores at end of treatment in a repeated measurements model. The model will be a mixed model for repeated measurements including at least time point, treatment group, the interaction between time point and treatment, sex, age and severity as fixed factors, baseline PANSS score as covariate and subject as random intercept factor. An cAR(1) structure will be used to model the residual covariance matrix. The primary analysis will be to test the contrast between prednisolon addition and control at 6 weeks follow-up. This will be presented with a 95% confidence interval for the difference between the treatment arms.

**10.2 Secondary study parameter(s)**

The secondary analysis will include the PANSS scores at 6 months follow-up, BACS scores at end of treatment and at 6 months follow-up in a repeated measurements model. The model will be a mixed model for repeated measurements including at least time point, treatment group, the interaction between time point and treatment, sex, age and severity as fixed factors, baseline BACS score as covariate and subject as random intercept factor. An cAR(1) structure will be used to model the residual covariance matrix. The primary analysis will be to test the contrast between prednisolone addition and control at 6 end of treatment follow-up. This will be presented with a 95% confidence interval for the difference between the treatment arms.

The secondary analyses on other continuous measures will be similar to the PANSS analyses. Secondary analyses on dichotomous data (e.g. on presence of metabolic syndrome) will be conducted using logistic regression analysis.

## 10.3 Other study parameters

The other analyses on continuous measures will also be similar to the primary analyses (but again with a different number of time points). Safety data: Incidences (number and % of subjects with at least one occurrence) of key SAEs and AEs will be presented per group. For exploratory purposes, confidence intervals comparing both groups will be provided.

## 10.4 Prediction model for response to prednisolone

An important aim of this study is to predict treatment response to prednisolone based on baseline blood markers. For this analysis we will use the arbitrary cut-off of 25% improvement in PANSS rating as a criterion for treatment response. A support vector machine (SVM) will be used to predict outcome (ie response or no response) on the basis of > 100 blood factors measured at baseline

# ETHICAL CONSIDERATIONS

##

## 11.1 Regulation statement

The study will be conducted in accordance with this protocol as well as the principles of the Declaration of Helsinki (64^th^ WMA general assembly; Fortaleza, Brazil, October 2013), the ICH-GCP guidelines and other applicable laws and regulations.

## 11.2 Recruitment and consent

Patients diagnosed with schizophrenia, schizoaffective or schizophreniform disorder (DSM-IV 295.*) or psychosis NOS (298.9) will be invited to participate through the psychiatry department of participating centers as well as the patient’s and family associations. Participation in the study is preceded by an informative meeting with the study investigator, during which the participant must be informed about the entire course of the study, potential individual benefits and personal risks. Here it must be emphasized that participation is absolutely voluntary. Patients are given sufficient time to read all the provided information, counsel partners or relatives, and clarify any questions with the investigator or the independent physician. Participation requires written consent before any (screening) procedure takes place. This consent can be revoked at any time without citing reasons and without any consequences for their clinical treatment. A copy of the consent form and patient information will be given to the participant. Patients with schizophrenia and related disorders may at times have reduced capacity to provide an informed consent because of disorder-related issues such as thought disorders or cognitive impairments. To ensure the capacity for providing informed consent, all eligible patients must be deemed able to provide informed consent by their treating physician, psychologist or psychiatrist. Furthermore, study physicians and psychiatrists will provide the detailed information about the project and also independently assess the capacity for providing informed consent. After informed consent is obtained, the participating centers will perform the first screening blood sample including high-sensitivity CRP.

## 11.3 Benefits and risks assessment, group relatedness

There is ample clinical experience with prednisolone and its safety profile is well known. Use of prednisolone is associated with a certain risk of side effects. Because of these risks, patients will only be included if they are overall physically healthy and relevant health aspects are monitored throughout the study. The time of exposure to prednisolone is minimized to 6 weeks to prevent side-effects such as diabetes, Cushing syndrome and osteoporosis, which are associated to chronic use. Apart from the somatic side-effects, prednisolone has been reported to induce psychiatric symptoms such as depression, confusion or psychosis. These psychiatric side-effects are dose dependent and the odds ratio's for these side-effects are <2 for dose <40mg. The risk for (exacerbation of) psychosis is expected to be lower in our sample as patients are using antipsychotic medication. Nevertheless, because of this risk for psychiatric side effects, patients will be interviewed at weekly intervals to assess symptom severity, mood, global functioning and suicidal ideation. In case of worsening of depression, psychosis or the development of suicidal ideas, study medication will be tapered off immediately (see stopping rules p.13.3).

Blood draws are associated with negligible and known risks (e.g. irritation). The placebo condition is not withheld any standard treatment. Overall, it is acknowledged that certain risks may be present, however the study procedures to monitor safety and wellbeing are performed by physicians, in such frequency that, in our opinion, actual risks will be identified swiftly and appropriately acted upon. The extra risk associated with study participation is balanced by considerable possible benefit. One benefit of participation is that routine care consists of less extensive monitoring of symptoms change and function compared to the current trial, so all patients may benefit from the thorough examinations during study participation. In the whole group treated with prednisolone, we expect a slowing of progression of negative and cognitive symptoms as compared to patients who are not immune modulated. Apart from these potential personal benefits for the participants, this study may have a large impact for the treatment of schizophrenia patients in general. When we succeed to define a subgroup with advantageous response to immune suppression, these baseline blood parameters can be used in larger population to identify patients to be treated with the combination of antipsychotic and immune suppressive therapy. If prednisolone proves to be neuroprotective in the whole group of treated patients, this will provide the proof of concept for the addition of anti-inflammatory therapy for patients with recent onset schizophrenia.

These burden and risks are considered acceptable while the results may give raise to a new line of scientific research as well as treatment options for recent-onset patients. Therefore, offering participation to selected patients appears to be justified.

## 11.4 Compensation for injury

The sponsor/investigator has liability insurance in accordance with the Product Liability Act in the Drug Insurance. Any damage including death or physical injury, caused by this research project, is covered by this insurance. The insurance covers for injury to a maximum sum of NOK 80 millions for injuries in one and the same calendar year, and NOK 100 million per serial injury. In case of injury, this must be reported to the insurer mentioned below as soon as possible after occurrence of this injury. The insurer’s name is: Legemiddelansvarsforeningen v/ advokat Gunnar Sørlie, Advokatfirmaet BA-HR DA, Postboks 1524 Vika, 0117 Oslo.

## 11.5 Incentives (if applicable)

Participants will receive a monetary compensation for time investments and travel expenses of NOK 200 for each visit during the study. If subjects wish to abort the study before completion, payment will be in proportion to the part of the procedure that has been completed.

# 12. ADMINISTRATIVE ASPECTS, MONITORING AND PUBLICATION

## 12.1 Handling and storage of data and documents

Privacy laws and regulations will be adhered to during the complete study. The collection and processing of participants’ personal information will be limited to what is necessary to ensure the study’s scientific practicability, the evaluation of efficacy, adherence, side effects and the investigational product’s safety. Information collected about participants during this clinical investigation will be treated confidentially. The investigator or her co-workers will collect data and transfer it without recording the patient’s name or date of birth. Instead data will be coded with a participant identification number.

The file with the key to the code will be managed by one person. The source documents will be kept in a locked file cabinet in the office of the study coordinator with limited access of the research personnel. In accordance with national laws and guidelines and the specifications of the ICH-GCP guidelines, the investigators are obligated to archive all documents pertaining to the study for the legally required time period.

The acquired data and examination results will be entered into an electronic case record form (eCRF) that is accessible via the internet. Investigators will receive personal user names and passwords for this purpose, and data will be encrypted for transfer. It will be agreed before the start of the study which documents serve as source documents for all data entered into the eCRF. For more detail on the handling and storage of collected data can be found in the separate SOP on Data Management.

## 12.2 Monitoring and Quality Assurance

Associated investigators will be carefully selected and comprehensively informed and trained regarding Good Clinical Practice (GCP), all study procedures and the required examinations and documentation. The quality of data acquisition will be confirmed by regular monitoring visits. An external monitor will be appointed by Haukeland University Hospital. The monitor is independent from the study team and is not involved in the inclusion of participants and the design and implementation of the study. All monitoring activities will be in line with national laws and guidelines and the specifications of the ICH-GCP guidelines.

The study monitor will visit the study site at regular intervals to monitor the execution of the study. The monitor will have access to all documents that are needed to perform his/her task according to the above mentioned guidelines. The monitor will check whether requirements to conduct the study are met and study procedures are followed correctly, and will check the study site’s documentation, the participants’ source data, eCRF entries, and the correct maintenance of the Investigator Site File. Investigators will permit trial-related monitoring, audits, ERB reviews and regulatory inspections, providing direct access to source data and study documents.

## 12.3 Amendments

A ‘substantial amendment’ is defined as an amendment to the terms of the METC application, or to the protocol or any other supporting documentation, that is likely to affect to a significant degree:

1. the safety or physical or mental integrity of the subjects of the trial;
2. the scientific value of the trial;
3. the conduct or management of the trial; or
4. the quality or safety of any intervention used in the trial.

All substantial amendments will be notified to the METC and to the competent authority.

Non-substantial amendments will not be notified to the accredited METC and the competent authority, but will be recorded and filed by the sponsor.

## 12.4 Annual progress report

The sponsor/investigator will submit a summary of the progress of the trial to the accredited METC once a year. Information will be provided on the date of inclusion of the first subject, numbers of subjects included and numbers of subjects that have completed the trial, serious adverse events/serious adverse reactions, other problems, and amendments.

## 12.5 End of study report

The sponsor will notify the accredited METC and the competent authority of the end of the study within a period of 90 days. The end of the study is defined as the last patient’s last visit.

In case the study is ended prematurely, the sponsor will notify the accredited METC and the competent authority within 15 days, including the reasons for the premature termination.

Within one year after the end of the study, the investigator/sponsor will submit a final study report with the results of the study, including any publications/abstracts of the study, to the accredited METC and the Competent Authority.

## 12.6 Public disclosure and publication policy

The results of the study will be submitted for publication in international peer-reviewed journals adhering to applicable privacy laws and regulations. Publication strategy will be determined by the principal investigator. No treatment group information will be made available until after study completion.

# 13. STRUCTURED RISK ANALYSIS

## 13.1 Potential issues of concern

a. Level of knowledge about mechanism of action

Prednisolone is a (semi)synthetic derivative of cortisol, a hormone synthesized by the adrenal cortex. Within the group of corticosteroids it is classified by chemical structure to the hydrocortisone group (type A) which are short to medium acting agents with predominantly glucocorticoid properties. Although not as potent as the mineralocorticoid aldosterone, nonfluorinated glucocorticoids (prednisolone and methylprednisolone) do have some effects on water and electrolyte balance, enhancing potassium excretion and sodium retention primarily due to their activity in the kidneys. Prednisolone is a δ-corticoid as well as prednisone and methylprednisolone and their glucocorticoid effect is 4 to 5 times as powerful as natural cortisol (http://www.merckmanuals.com; http://legemiddelhandboka.no/Legemidler/43981?expand=1)

Glucocorticoids diffuse through the cell membrane as lipophilic molecules after which cytoplasmic binding of the glucocorticoidreceptor (GCR) takes place. Prednisolone irreversibly binds with (GCR) alpha and beta for which they have a high affinity. Prednisolone can activate and influence biochemical behavior of most cells. Glucocorticoids have various effects on the immune system at several levels and are both anti-inflammatory and immunosuppressive when administered therapeutically (Buttgereit, 2000). Their therapeutic effects are considered to be mediated by four different mechanisms of action: the classical genomic mechanism of action caused by the cytosolic glucocorticoid receptor (cGCR); secondary non-genomic effects which are also initiated by the cGCR; membrane-bound glucocorticoid receptor (mGCR)-mediated non-genomic effects; non-specific, non-genomic effects caused by interactions with cellular membranes. Non-genomic effects are mostly seen in higher dosed glucocorticosteroids. The genomic effects are established by a process in which the GCRcomplex diffuses from cytoplasm to nucleus and there regulates the transcription of genes. DNA is read and messenger RNA is formed after which protein production occurs. The classical, genomic mechanism of GC-action can be divided into two processes: “transrepression” and “transactivation” (Stahn et al., 2007). In transrepression the GCRcomplex binds to transcription factors such as nuclear factor kappaB (NF-kB) and activator protein 1, by which transcription is inhibited. This results in diminished synthesis of proinflammatory cytokines (Huisman et al., 2006). Transrepression is responsible for a large number of desirable anti-inflammatory and immune modulating effects. Transactivation however is associated with frequently occurring side effects as well as with some immunosuppressive activities (Stahn et al., 2007). In this process the GCRcomplex of genes coding for metabolic and endocrine proteins are stimulated, for instance inducing gluconeogenesis (Huisman et al., 2006). Through the regulation of gene suppression prednisolone leads to systematic suppression of inflammation and immune response. Specifically, its anti-inflammatory and immunosuppressive effect is established by the inhibition of leukocyte production, the migration of leukocytes to the site of inflammation and by inhibiting leukocyte functioning for instance by decreasing synthesis of proinflammatory cytokines and prostaglandins.

b. Previous exposure of human beings with the test product(s) and/or products with a similar biological mechanism

Glucocorticoids like prednisolone have been successfully used for over 50 years in the treatment of a wide range of inflammatory and non-inflammatory diseases. It is proven to be effective and side-effects have been extensively studied.

c. Can the primary or secondary mechanism be induced in animals and/or in *ex-vivo* human cell material?

The mechanism of glucorticosteriods is similar in various animals, as it is widely used as treatment for inflammatory conditions in veterinary settings. However, in the absence of a valid animal model for schizophrenia, our hypotheses cannot be studied in animal models or in ex-vivo settings.

d. Selectivity of the mechanism to target tissue in animals and/or human beings

Prednisolone irreversibly binds with GCR alpha and beta for which they have a high affinity. These receptors are found in virtually all tissues including brain tissue, with variable numbers between 3000 and 10000 per cell, depending on the tissue involved. Prednisolone is, in contrast to most glucocorticosteriods, able to cross the blood-brain barrier.

e. Analysis of potential effect

After oral administration, approximately one third of the prednisolone blood level is measured in the CNS. We therefore consider a minimal dose of 40mg necessary to obtain satisfactory immune suppression in the brain.

Some indication for a dose-response relationship was found with regards to side effects of glucocorticoids in general (McDonough et al., 2008) although this was not demonstrated in other studies. Potentially harmful effects can be found in the psychiatric domain; neuropsychiatric adverse events and behavioral disturbances related to corticosteroid use have been reported (Hoes et al., 2009, Fardet et al., 2012). Prednisolone can trigger or aggravate (hypo)mania, psychosis and depression. These psychiatric side effects are dose-dependent. One study reported psychiatric side effects that were largely observed at doses of more than 80mg per day (Brown, 2009). The Boston Collaborative Drug Surveillance Program (1972) reported an incidence of 1.3% for psychiatric side effects during prednisolone use in daily dosages lower than 40mg. A more recent study showed an increased risk of 1.6 for depression and 3.2 for mania with doses higher than 40mg, whereas risk for delirium, confusion or disorientation and panic disorder were already increased for doses higher than 10mg (hazard ratio of 1.3 and 3.4, respectively (Fardet et al., 2012). An increased risk of 6.9 for suicidal behaviour was observed in patients treated with corticosteroids compared to patients with a similar medical condition who were not treated with corticosteroids (Fardet et al. 2012).

For non-psychiatric side effects, there remains debate on the true incidence since it is difficult to separate the side effects from the outcome of the underlying disease. Dose-effect relationships regarding side effects have been extensively studied. In low doses (max. 10mg/day), medium to long term use (≥ 2 years) of prednisolone was found to have limited toxicity compared to placebo in a meta-analysis on rheumatoid arthritis (Ravindran et al., 2009). Disease-specific variation in adverse event occurrence has been reported. A meta-analysis of low (< 7.5mg) to medium (≤ 30mg) dosed glucocorticoids in several inflammatory disorders found a mean adverse event rate of 150 events per 100 patient-years, after exposure to glucocorticoids of at least one month (Hoes et al., 2009). Occurrence of adverse effects ranged from 43/100 patient-years in rheumatic arthritis to 555/100 patient-years in Inflammatory Bowel Disorder, although the differences in study designs partly explained these variations. Comparison of low and medium dosages did not show dose dependency of any specific adverse event.

Osteoporosis is a well-established side effect of (chronic) glucocorticoid use. A meta-analysis by van Staa et al. (van Staa et al., 2002) concludes that corticosteroid therapy is associated with a dose related increase of risk of fracture and bone mineral density (BMD), which may be substantially reversible after discontinuation of corticosteroids and are independent of underlying disease, age and sex. In this meta-analysis it was estimated that a cumulative dose of 13.9 gram prednisolone would correspond to an expected BMD loss of 4.7% at the spine and 6.1% at the hip. The onset of bone loss is rapid within the first months of starting corticosteroids, slowing down after one year of therapy (van Staa et al., 2002). Therefore it is advised to prescribe vitamin D and calcium for patients on long courses of glucocorticoid (McDonough et al., 2008). Another common adverse effect of corticosteroids is symptoms of Cushing’s syndrome. Most recognizable is a Cushingoid habitus where adipose tissue accumulates in facial, dorsocervical and abdominal regions, whereas subcutaneous fat thickness is reduced in the limbs. This is also called corticosteroid-induced lipodystrophy. Its prevalence is variable, with limited data-availability on risk factors and dose-dependency (Fardet et al., 2007), however in several studies in short term use of corticosteroids prevalence ranging from 15%-40% were seen with dosages of 10-30mg or mean 23mg prednisone equivalent (Bar-Meir et al., 1998, Campieri et al., 1997, Shubin, 1965). Lipodystrophy is usually reversible when this emerged during a treatment with prednisone equivalent daily dosage of <10mg/day (Fardet et al., 2007). No such adverse effects have been observed in a corresponding study already ongoing in the Netherlands, which have included 20 patients thus far (Iris Sommer, Principal Investigator, the **Prednisolone addition for patients with recent-onset psychotic disorder** study (Eudra CT-number 2014-000520-14), Personal communication). Risk of cardiovascular events is increased in corticosteroid use. Wei (Wei et al., 2004) reported an odds ratio of 2.56 for total cardiovascular events in patients using > 7.5mg systemic prednisolone. The risk was significant for heart failure, mycardial infarction and ischaemic stroke. Cases of arrythmias and sudden death are rare. Patients with a glucocorticoid induced lipodystrophy have a threefold higher risk of cardiovascular events (Fardet et al., 2012).

Hypertension is a well-known adverse effect in steroid use, previously thought to be caused by mineralcorticoid capacities, but also seen in glucocorticosteroid use. Cardiovascular risk is increased in corticosteroid use, however its precise prevalence is not known; an odds ratio of 1.3-2.6 was reported by Fardet (Fardet et al., 2007). Increase of glucose serum levels is also well known in glucocorticoid use. Although glucocorticoid related hyperglycemia is dose dependent, it is also seen in low doses (Da Silva et al., 2006). Davenport et al. (Davenport et al., 2011) studied short-term administration of high dosed corticosteroid (600mg hydrocortisone) given prior to administration of radiologic contrast medium, which caused development of a brief and reversible hyperglycemia.

Head Consultant, MD, (seksjonsoverlege) Hrafnkell Thordarson at the Section of Endocrinology, Department of Medicine, Haukeland University Hospital, has been consulted for expert advice regarding risk considerations related to potential suppression of the adrenal cortical function by the prednisolone treatment regimen in the study. Dr. Thordarson states that gradual down-titration over 6 weeks with 5 mg the final week is regarded a safe approach and is common in clinical practice. The patients should be informed about the possibility of reduced adrenal cortical function for a few days following discontinuation of prednisolone, such as laxity and dizziness, including orthostatic dizziness. No biochemical/ laboratory assessments are indicated (Hrafnkell Thordarson, Personal communication).

f. Pharmacokinetic considerations

After oral administration most glucocorticoids are resorbed within 30 minutes. In man, absorption of prednisolone is good. Maximum plasma concentration in prednisolone is reached in 1 to 2 hours. In plasma glucocorticoids are bound to proteins, transcortine and albumin. Protein-bound glucocorticoid is biologically non-active. One third circulates as free glucocorticoid and is biologically active. Prednisolone is mainly metabolised in the liver and has a usual plasma half-life of 2-3 hours. Prednisolone is eliminated via urine consisting of both free and conjugated metabolites and a portion of unchanged prednisolone.

g. Study population

Patients included in this study are expected to be in normal physical health. Patients with conditions meeting the contraindications and/or warnings for use included in the prednisolone Summary of Product Characteristics will be excluded from the study. Patients with schizophrenia, schizophreniform or schizoaffective disorder, or psychosis NOS, are eligible for the study. Acute psychotic symptoms are neither an inclusion nor an exclusion criterion, therefore it is not a requirement that patients are mentally stable, as long as they are mentally competent. They are required to be on a stable treatment of antipsychotics for a specific period of time. The in- and exclusion criteria will prevent the inclusion of children and pregnant women. Women of childbearing potential are allowed to participate but have to utilize an acceptable contraceptive in case of sexual intercourse during the study.

h. Interaction with other products

Due to the risk of interactions, the concurrent use of specific products is not allowed in the current study. Concurrent use of enzyme inducing medication such as carbamazepine, riphampicine, primidone, barbiturates and phenytoine is not allowed. In addition, the use of HAART (both HIV protease inhibitors and (non)-nucleoside reverse transcriptase inhibitors), especially efavirenz, ritonavir and lopinavir, is forbidden. Current use of telaprevir and boceprevir in treatment of Hepatitis C as well as the administration of live vaccines are not allowed. Finally, NSAID use is not allowed.

Concurrent use of non kalium-sparing antihypertensive drugs may cause an increased risk of hypokalemia. Caution should be exercised when prednisolone is combined with oral anticoagulants (coumarine derivates) due to risk of increased coagulation and potential clotting.

i. Predictability of effect

As the current trial concerns a ‘proof of concept’ study, there is no data available on biomarkers that can predict the effects of immune-modulation on psychosis symptoms and cognitive functioning. Through this study we intend to establish whether there is an effect at all, and if this is the case, post hoc analyses can be employed to assess the presence of ay biomarkers.

j. Can effects be managed?

The prevalence of side-effects needing intervention is expected to be low. Wellbeing of patients, physically as well as mentally, is closely monitored by experienced physicians. A set of stopping rules are defined (refer to next section) to ensure patient’s safety. In the majority of cases where the patient should not continue in the study due to side effects, prednisolone can be tapered down causing the side effect to abate. If needed, collaboration with applicable specialist (e.g. endocrinologists) is immediately sought. In emergency situations, a 24 hour / 7 days a week unblinding procedure is in place. For psychiatric events, prompt treatment will be offered at psychiatry departments of the participating centres. We expect a maximum of 10 patients to be in the active treatment phase of the study simultaneously, which can easily be managed by the study team.

## 13.2 Synthesis

The well-established efficacy of prednisolone to lower the immune response is counterbalanced by a considerable amount of side-effects, both physical and psychiatric. The most common somatic side effects are hyperglycaemia (Caplan et al., 2017b) and loss of bone mass (van Staa et al., 2002, Caplan et al., 2017a). Even though the duration of glucocorticoid exposure will be relatively short, these risks are managed pro-actively. To prevent corticosteroid induced diabetes mellitus (DM), baseline screening for hyperglycaemia will take place. Patients with initial random glucose levels exceeding 200 mg/dL, or who have pre-existing DM, will be excluded. As steroid-induced DM is dependent on body weight (Caplan et al., 2017b), patients with a BMI exceeding 27.5 will not be included in the study. During the trial, blood glucose and cell counts will be assessed repeatedly. To prevent loss of bone mass, we will provide calcium supplementation (calciumcarbonate 500mg daily) and vitamin D supplementation (colecalciferol 400ie daily) to both groups during the treatment phase. Patients will be observed so as not to overlook Cushingoid symptoms, although the limited extent of exposure is expected to be too short for the development of these symptoms.

With regards to cardiovascular side effects, blood pressure will be measured repeatedly during this study. Patients are informed of cardiac symptoms like chest pains and are instructed to consult the study physician immediately if they occur.

In the psychiatric domain, prednisolone can trigger or aggravate (hypo)mania, psychosis and depression. The PANSS and CDSS interview will be used to assess current presence and severity of, as well as change in, psychotic and depressive symptoms. Prednisolone is used as augmentation next to existent antipsychotic treatment, therefore reducing the risk for (exacerbation of) psychotic symptoms. Patients will be visited weekly by a trial physician who will assess suicidality. When patients develop suicidal thoughts, study medication will be discontinued and they will be visited daily or admitted to a ward if necessary. Corticosteroid-induced mood alterations have been shown to be reversible with dose reduction or discontinuation of treatment (Brown et al., 2009).

Overall, by applying elaborate in- and exclusion criteria, implementing appropriate pro-active safety measures for eligible patients, limiting the prednisolone exposure to 6 weeks and applying the stopping rules, the fact that all included patients are treated with antipsychotics, the risks are manageable and acceptable in our opinion. Safety of the study will be judged by an independent Data Safety Monitoring Board (DSMB), consisting of experts on both schizophrenia and immune dysregulation, who will meet each time after 10 additional patients are recruited (refer to section 9.5).

Most importantly, a corresponding study is already ongoing in the Netherlands (Eudra CT-number 2014-000520-14), and a safety report is available for the first 15 patients included (see Supplement 1). In conclusion, within the current study 15 patients have been exposed to prednisolone or placebo. Overall there is a reduction of psychotic symptoms. None of the patients showed a relapse, except one patient who had stopped antipsychotic medication during the trial (reported as an SAE). This was the only SAE since the start of the study.

There were no Suspected Unexpected Serious Adverse Reactions (SUSARs).

No additional changes in the study design or procedures were executed due to a need to increase patient safety.

This study will have a high impact as it defines whether or not modulation of the immune system is a useful intervention for schizophrenia. If this study shows an improvement in symptom severity or cognition, it will strengthen the case for immune-modulatory treatment and encourage further research into this direction. An obvious next step would be to investigate which specific component can provide optimal immune modulation with minimal side-effects in patients with schizophrenia.

On the other hand, if there are no beneficial effects of potent immune suppression identified in this study, the chances are small that more specific and weaker anti-inflammatory agents will provide effective treatments for people with schizophrenia and further research should better focus on other promising treatment strategies.

## 13.3 Stopping rules - individuals

Several events that may jeopardize the patient's health will prompt clinicians to end the study, and start tapering the patient off the study medication immediately in line with the treatment guidelines for Inflammatory Bowel Diseases. These events include the patient developing: a blood glucose exceeding 11.1 mmol/L (200mg/dL), suicidal ideations, the PANSS positive subscores which increase by 10 or more points without a clear reason (i.e. medication non-adherence), the PANSS item G6 exceeding a score of 4, the need for coercive treatment, pregnancy, or a systematic infectious disease.

**13.4 Stopping rules – the study as a whole (See also Section 8.7)**

The study as a whole can be terminated prematurely in case of a change in the risk profile of prednisolone, making it necessary to re-assess the use of the investigational product. The monitoring and advice of the DSMB will be central in this assessment.

In case the entire study is discontinued, patients will be followed-up and/or treated as usual in normal daily practice, depending on the severity of the changes in risk profile and the extent to which this increase in risk is applicable for individual patients.

# REFERENCES

1972. Acute adverse reactions to prednisone in relation to dosage. *Clin Pharmacol Ther,* 13**,** 694-8.

ADDINGTON, D., ADDINGTON, J. & SCHISSEL, B. 1990. A depression rating scale for schizophrenics. *Schizophr Res,* 3**,** 247-51.

AGERBO, E., BYRNE, M., EATON, W. W. & MORTENSEN, P. B. 2004. Marital and labor market status in the long run in schizophrenia. *Arch Gen Psychiatry,* 61**,** 28-33.

ARIAS, I., SORLOZANO, A., VILLEGAS, E., DE DIOS LUNA, J., MCKENNEY, K., CERVILLA, J., GUTIERREZ, B. & GUTIERREZ, J. 2012. Infectious agents associated with schizophrenia: a meta-analysis. *Schizophr Res,* 136**,** 128-36.

BAR-MEIR, S., CHOWERS, Y., LAVY, A., ABRAMOVITCH, D., STERNBERG, A., LEICHTMANN, G., RESHEF, R., ODES, S., MOSHKOVITZ, M., BRUCK, R., ELIAKIM, R., MAOZ, E. & MITTMANN, U. 1998. Budesonide versus prednisone in the treatment of active Crohn's disease. The Israeli Budesonide Study Group. *Gastroenterology,* 115**,** 835-40.

BECHTER, K., REIBER, H., HERZOG, S., FUCHS, D., TUMANI, H. & MAXEINER, H. G. 2010. Cerebrospinal fluid analysis in affective and schizophrenic spectrum disorders: identification of subgroups with immune responses and blood-CSF barrier dysfunction. *J Psychiatr Res,* 44**,** 321-30.

BELLACK, A. S., GREEN, M. F., COOK, J. A., FENTON, W., HARVEY, P. D., HEATON, R. K., LAUGHREN, T., LEON, A. C., MAYO, D. J., PATRICK, D. L., PATTERSON, T. L., ROSE, A., STOVER, E. & WYKES, T. 2007. Assessment of community functioning in people with schizophrenia and other severe mental illnesses: a white paper based on an NIMH-sponsored workshop. *Schizophr Bull,* 33**,** 805-22.

BENROS, M. E., NIELSEN, P. R., NORDENTOFT, M., EATON, W. W., DALTON, S. O. & MORTENSEN, P. B. 2011. Autoimmune diseases and severe infections as risk factors for schizophrenia: a 30-year population-based register study. *Am J Psychiatry,* 168**,** 1303-10.

BORM, G. F., FRANSEN, J. & LEMMENS, W. A. 2007. A simple sample size formula for analysis of covariance in randomized clinical trials. *J Clin Epidemiol,* 60**,** 1234-8.

BROWN, A. S. & DERKITS, E. J. 2010. Prenatal infection and schizophrenia: a review of epidemiologic and translational studies. *Am J Psychiatry,* 167**,** 261-80.

BROWN, E. S. 2009. Effects of glucocorticoids on mood, memory, and the hippocampus. Treatment and preventive therapy. *Ann N Y Acad Sci,* 1179**,** 41-55.

BUTTGEREIT, F. 2000. Mechanisms and clinical relevance of nongenomic glucocorticoid actions. *Z Rheumatol,* 59 Suppl 2**,** II/119-23.

CAHN, W., VAN HAREN, N. E., HULSHOFF POL, H. E., SCHNACK, H. G., CASPERS, E., LAPONDER, D. A. & KAHN, R. S. 2006. Brain volume changes in the first year of illness and 5-year outcome of schizophrenia. *Br J Psychiatry,* 189**,** 381-2.

CAMPIERI, M., FERGUSON, A., DOE, W., PERSSON, T. & NILSSON, L. G. 1997. Oral budesonide is as effective as oral prednisolone in active Crohn's disease. The Global Budesonide Study Group. *Gut,* 41**,** 209-14.

CAPLAN, A., FETT, N., ROSENBACH, M., WERTH, V. P. & MICHELETTI, R. G. 2017a. Prevention and management of glucocorticoid-induced side effects: A comprehensive review: A review of glucocorticoid pharmacology and bone health. *J Am Acad Dermatol,* 76**,** 1-9.

CAPLAN, A., FETT, N., ROSENBACH, M., WERTH, V. P. & MICHELETTI, R. G. 2017b. Prevention and management of glucocorticoid-induced side effects: A comprehensive review: Gastrointestinal and endocrinologic side effects. *J Am Acad Dermatol,* 76**,** 11-16.

CHAN, M. K., GUEST, P. C., LEVIN, Y., UMRANIA, Y., SCHWARZ, E., BAHN, S. & RAHMOUNE, H. 2011. Converging evidence of blood-based biomarkers for schizophrenia: an update. *Int Rev Neurobiol,* 101**,** 95-144.

CURTIS, J. R., WESTFALL, A. O., ALLISON, J., BIJLSMA, J. W., FREEMAN, A., GEORGE, V., KOVAC, S. H., SPETTELL, C. M. & SAAG, K. G. 2006. Population-based assessment of adverse events associated with long-term glucocorticoid use. *Arthritis Rheum,* 55**,** 420-6.

DA SILVA, J. A., JACOBS, J. W., KIRWAN, J. R., BOERS, M., SAAG, K. G., INES, L. B., DE KONING, E. J., BUTTGEREIT, F., CUTOLO, M., CAPELL, H., RAU, R. & BIJLSMA, J. W. 2006. Safety of low dose glucocorticoid treatment in rheumatoid arthritis: published evidence and prospective trial data. *Ann Rheum Dis,* 65**,** 285-93.

DAVENPORT, M. S., COHAN, R. H., KHALATBARI, S., MYLES, J., CAOILI, E. M. & ELLIS, J. H. 2011. Hyperglycemia in hospitalized patients receiving corticosteroid premedication before the administration of radiologic contrast medium. *Acad Radiol,* 18**,** 384-90.

DAVIDSON, M., GALDERISI, S., WEISER, M., WERBELOFF, N., FLEISCHHACKER, W. W., KEEFE, R. S., BOTER, H., KEET, I. P., PRELIPCEANU, D., RYBAKOWSKI, J. K., LIBIGER, J., HUMMER, M., DOLLFUS, S., LOPEZ-IBOR, J. J., HRANOV, L. G., GAEBEL, W., PEUSKENS, J., LINDEFORS, N., RIECHER-ROSSLER, A. & KAHN, R. S. 2009. Cognitive effects of antipsychotic drugs in first-episode schizophrenia and schizophreniform disorder: a randomized, open-label clinical trial (EUFEST). *Am J Psychiatry,* 166**,** 675-82.

DEN UYL, D., VAN RAALTE, D. H., NURMOHAMED, M. T., LEMS, W. F., BIJLSMA, J. W., HOES, J. N., DIJKMANS, B. A. & DIAMANT, M. 2012. Metabolic effects of high-dose prednisolone treatment in early rheumatoid arthritis: balance between diabetogenic effects and inflammation reduction. *Arthritis Rheum,* 64**,** 639-46.

DICKERSON, F., STALLINGS, C., ORIGONI, A., BORONOW, J. & YOLKEN, R. 2007. C-reactive protein is associated with the severity of cognitive impairment but not of psychiatric symptoms in individuals with schizophrenia. *Schizophr Res,* 93**,** 261-5.

DICKERSON, F., STALLINGS, C., ORIGONI, A., VAUGHAN, C., KHUSHALANI, S., YANG, S. & YOLKEN, R. 2013. C-reactive protein is elevated in schizophrenia. *Schizophr Res,* 143**,** 198-202.

DICKERSON, F., STALLINGS, C., ORIGONI, A., VAUGHAN, C., KHUSHALANI, S. & YOLKEN, R. 2012. Additive effects of elevated C-reactive protein and exposure to Herpes Simplex Virus type 1 on cognitive impairment in individuals with schizophrenia. *Schizophr Res,* 134**,** 83-8.

DOORDUIN, J., DE VRIES, E. F., WILLEMSEN, A. T., DE GROOT, J. C., DIERCKX, R. A. & KLEIN, H. C. 2009. Neuroinflammation in schizophrenia-related psychosis: a PET study. *J Nucl Med,* 50**,** 1801-7.

DREXHAGE, R. C., HOOGENBOEZEM, T. A., COHEN, D., VERSNEL, M. A., NOLEN, W. A., VAN BEVEREN, N. J. & DREXHAGE, H. A. 2011. An activated set point of T-cell and monocyte inflammatory networks in recent-onset schizophrenia patients involves both pro- and anti-inflammatory forces. *Int J Neuropsychopharmacol,* 14**,** 746-55.

DREXHAGE, R. C., VAN DER HEUL-NIEUWENHUIJSEN, L., PADMOS, R. C., VAN BEVEREN, N., COHEN, D., VERSNEL, M. A., NOLEN, W. A. & DREXHAGE, H. A. 2010. Inflammatory gene expression in monocytes of patients with schizophrenia: overlap and difference with bipolar disorder. A study in naturalistically treated patients. *Int J Neuropsychopharmacol,* 13**,** 1369-81.

FAN, X., PRISTACH, C., LIU, E. Y., FREUDENREICH, O., HENDERSON, D. C. & GOFF, D. C. 2007. Elevated serum levels of C-reactive protein are associated with more severe psychopathology in a subgroup of patients with schizophrenia. *Psychiatry Res,* 149**,** 267-71.

FARDET, L., KASSAR, A., CABANE, J. & FLAHAULT, A. 2007. Corticosteroid-induced adverse events in adults: frequency, screening and prevention. *Drug Saf,* 30**,** 861-81.

FARDET, L., PETERSEN, I. & NAZARETH, I. 2012. Suicidal behavior and severe neuropsychiatric disorders following glucocorticoid therapy in primary care. *Am J Psychiatry,* 169**,** 491-7.

GARDINER, E., BEVERIDGE, N. J., WU, J. Q., CARR, V., SCOTT, R. J., TOONEY, P. A. & CAIRNS, M. J. 2012. Imprinted DLK1-DIO3 region of 14q32 defines a schizophrenia-associated miRNA signature in peripheral blood mononuclear cells. *Mol Psychiatry,* 17**,** 827-40.

GREEN, M. F. 1996. What are the functional consequences of neurocognitive deficits in schizophrenia? *Am J Psychiatry,* 153**,** 321-30.

GREEN, M. F., KERN, R. S., BRAFF, D. L. & MINTZ, J. 2000. Neurocognitive deficits and functional outcome in schizophrenia: are we measuring the "right stuff"? *Schizophr Bull,* 26**,** 119-36.

HOES, J. N., JACOBS, J. W., VERSTAPPEN, S. M., BIJLSMA, J. W. & VAN DER HEIJDEN, G. J. 2009. Adverse events of low- to medium-dose oral glucocorticoids in inflammatory diseases: a meta-analysis. *Ann Rheum Dis,* 68**,** 1833-8.

HUISMAN, A. M., JACOBS, J. W., BUTTGEREIT, F. & BIJLSMA, J. W. 2006. [New developments in glucocorticoid therapy: selective glucocorticoid receptor agonists, nitrosteroids and liposomal glucocorticoids]. *Ned Tijdschr Geneeskd,* 150**,** 476-80.

IRVING, P. M., GEARRY, R. B., SPARROW, M. P. & GIBSON, P. R. 2007. Review article: appropriate use of corticosteroids in Crohn's disease. *Aliment Pharmacol Ther,* 26**,** 313-29.

JOHNSEN, E., FATHIAN, F., KROKEN, R. A., STEEN, V. M., JORGENSEN, H. A., GJESTAD, R. & LOBERG, E. M. 2016. The serum level of C-reactive protein (CRP) is associated with cognitive performance in acute phase psychosis. *BMC Psychiatry,* 16**,** 60.

KAHN, R. S. & KEEFE, R. S. 2013. Schizophrenia is a cognitive illness: time for a change in focus. *JAMA Psychiatry,* 70**,** 1107-12.

KARTERUD, S. P., G.; LOEVDAHL, H.; FRIIS, S. 1998. Global Assessment of Functioning - Split Version (S-GAF): Background and Scoring Manual. Oslo, Norway: Ullevaal University Hospital, Department of Psychiatry.

KAY, S. R., FISZBEIN, A. & OPLER, L. A. 1987. The positive and negative syndrome scale (PANSS) for schizophrenia. *Schizophr Bull,* 13**,** 261-76.

KEEFE, R. S., BILDER, R. M., DAVIS, S. M., HARVEY, P. D., PALMER, B. W., GOLD, J. M., MELTZER, H. Y., GREEN, M. F., CAPUANO, G., STROUP, T. S., MCEVOY, J. P., SWARTZ, M. S., ROSENHECK, R. A., PERKINS, D. O., DAVIS, C. E., HSIAO, J. K. & LIEBERMAN, J. A. 2007. Neurocognitive effects of antipsychotic medications in patients with chronic schizophrenia in the CATIE Trial. *Arch Gen Psychiatry,* 64**,** 633-47.

KEEFE, R. S., GOLDBERG, T. E., HARVEY, P. D., GOLD, J. M., POE, M. P. & COUGHENOUR, L. 2004. The Brief Assessment of Cognition in Schizophrenia: reliability, sensitivity, and comparison with a standard neurocognitive battery. *Schizophr Res,* 68**,** 283-97.

KEEFE, R. S. & HARVEY, P. D. 2012. Cognitive impairment in schizophrenia. *Handb Exp Pharmacol***,** 11-37.

KITCHEN, H., ROFAIL, D., HERON, L. & SACCO, P. 2012. Cognitive impairment associated with schizophrenia: a review of the humanistic burden. *Adv Ther,* 29**,** 148-62.

KNIGHT, J. G., MENKES, D. B., HIGHTON, J. & ADAMS, D. D. 2007. Rationale for a trial of immunosuppressive therapy in acute schizophrenia. *Mol Psychiatry,* 12**,** 424-31.

LAAN, W., GROBBEE, D. E., SELTEN, J. P., HEIJNEN, C. J., KAHN, R. S. & BURGER, H. 2010. Adjuvant aspirin therapy reduces symptoms of schizophrenia spectrum disorders: results from a randomized, double-blind, placebo-controlled trial. *J Clin Psychiatry,* 71**,** 520-7.

LAAN, W., SELTEN, J. P., GROBBEE, D. E., SMEETS, H., KAHN, R. S. & BURGER, H. 2007. Non-steroidal anti-inflammatory drugs and the risk of psychosis. *Eur Neuropsychopharmacol,* 17**,** 309-11.

LAAN, W., SMEETS, H., DE WIT, N. J., KAHN, R. S., GROBBEE, D. E. & BURGER, H. 2009. Glucocorticosteroids associated with a decreased risk of psychosis. *J Clin Psychopharmacol,* 29**,** 288-90.

LIEBERMAN, J. A., TOLLEFSON, G. D., CHARLES, C., ZIPURSKY, R., SHARMA, T., KAHN, R. S., KEEFE, R. S., GREEN, A. I., GUR, R. E., MCEVOY, J., PERKINS, D., HAMER, R. M., GU, H., TOHEN, M. & GROUP, H. S. 2005. Antipsychotic drug effects on brain morphology in first-episode psychosis. *Arch Gen Psychiatry,* 62**,** 361-70.

LOWENBERG, M., STAHN, C., HOMMES, D. W. & BUTTGEREIT, F. 2008. Novel insights into mechanisms of glucocorticoid action and the development of new glucocorticoid receptor ligands. *Steroids,* 73**,** 1025-9.

MCDONOUGH, A. K., CURTIS, J. R. & SAAG, K. G. 2008. The epidemiology of glucocorticoid-associated adverse events. *Curr Opin Rheumatol,* 20**,** 131-7.

MCGURK, S. R., MUESER, K. T., DEROSA, T. J. & WOLFE, R. 2009. Work, recovery, and comorbidity in schizophrenia: a randomized controlled trial of cognitive remediation. *Schizophr Bull,* 35**,** 319-35.

MISHARA, A. L. & GOLDBERG, T. E. 2004. A meta-analysis and critical review of the effects of conventional neuroleptic treatment on cognition in schizophrenia: opening a closed book. *Biol Psychiatry,* 55**,** 1013-22.

O'DONNELL, P. 2012. Cortical interneurons, immune factors and oxidative stress as early targets for schizophrenia. *Eur J Neurosci,* 35**,** 1866-70.

ORR, J. D. 2008. Statins in the spectrum of neurologic disease. *Curr Atheroscler Rep,* 10**,** 11-8.

RAISON, C. L., RUTHERFORD, R. E., WOOLWINE, B. J., SHUO, C., SCHETTLER, P., DRAKE, D. F., HAROON, E. & MILLER, A. H. 2013. A randomized controlled trial of the tumor necrosis factor antagonist infliximab for treatment-resistant depression: the role of baseline inflammatory biomarkers. *JAMA Psychiatry,* 70**,** 31-41.

RAVINDRAN, V., RACHAPALLI, S. & CHOY, E. H. 2009. Safety of medium- to long-term glucocorticoid therapy in rheumatoid arthritis: a meta-analysis. *Rheumatology (Oxford),* 48**,** 807-11.

REISS, A. B. & WIRKOWSKI, E. 2009. Statins in neurological disorders: mechanisms and therapeutic value. *ScientificWorldJournal,* 9**,** 1242-59.

ROBERTSON, D. A., HARGREAVES, A., KELLEHER, E. B., MORRIS, D., GILL, M., CORVIN, A. & DONOHOE, G. 2013. Social dysfunction in schizophrenia: an investigation of the GAF scale's sensitivity to deficits in social cognition. *Schizophr Res,* 146**,** 363-5.

SAHA, S., CHANT, D. & MCGRATH, J. 2007. A systematic review of mortality in schizophrenia: is the differential mortality gap worsening over time? *Arch Gen Psychiatry,* 64**,** 1123-31.

SATO, A., FUNDER, J. W., OKUBO, M., KUBOTA, E. & SARUTA, T. 1995. Glucocorticoid-induced hypertension in the elderly. Relation to serum calcium and family history of essential hypertension. *Am J Hypertens,* 8**,** 823-8.

SCHMIDT, S. C., HAMANN, S., LANGREHR, J. M., HOFLICH, C., MITTLER, J., JACOB, D. & NEUHAUS, P. 2007. Preoperative high-dose steroid administration attenuates the surgical stress response following liver resection: results of a prospective randomized study. *J Hepatobiliary Pancreat Surg,* 14**,** 484-92.

SCHMIDT, S. J., MUELLER, D. R. & RODER, V. 2011. Social cognition as a mediator variable between neurocognition and functional outcome in schizophrenia: empirical review and new results by structural equation modeling. *Schizophr Bull,* 37 Suppl 2**,** S41-54.

SCHWARZ, E., GUEST, P. C., STEINER, J., BOGERTS, B. & BAHN, S. 2012. Identification of blood-based molecular signatures for prediction of response and relapse in schizophrenia patients. *Transl Psychiatry,* 2**,** e82.

SCHWARZ, E., IZMAILOV, R., SPAIN, M., BARNES, A., MAPES, J. P., GUEST, P. C., RAHMOUNE, H., PIETSCH, S., LEWEKE, F. M., ROTHERMUNDT, M., STEINER, J., KOETHE, D., KRANASTER, L., OHRMANN, P., SUSLOW, T., LEVIN, Y., BOGERTS, B., VAN BEVEREN, N. J., MCALLISTER, G., WEBER, N., NIEBUHR, D., COWAN, D., YOLKEN, R. H. & BAHN, S. 2010. Validation of a blood-based laboratory test to aid in the confirmation of a diagnosis of schizophrenia. *Biomark Insights,* 5**,** 39-47.

SHEEHAN, D. V., LECRUBIER, Y., SHEEHAN, K. H., AMORIM, P., JANAVS, J., WEILLER, E., HERGUETA, T., BAKER, R. & DUNBAR, G. C. 1998. The Mini-International Neuropsychiatric Interview (M.I.N.I.): the development and validation of a structured diagnostic psychiatric interview for DSM-IV and ICD-10. *J Clin Psychiatry,* 59 Suppl 20**,** 22-33;quiz 34-57.

SHUBIN, H. 1965. Long term (five or more years) administration of corticosteroids in pulmonary diseases. *Dis Chest,* 48**,** 287-90.

SOMMER, I. E., DE WITTE, L., BEGEMANN, M. & KAHN, R. S. 2012. Nonsteroidal anti-inflammatory drugs in schizophrenia: ready for practice or a good start? A meta-analysis. *J Clin Psychiatry,* 73**,** 414-9.

SOMMER, I. E., VAN WESTRHENEN, R., BEGEMANN, M. J., DE WITTE, L. D., LEUCHT, S. & KAHN, R. S. 2014. Efficacy of anti-inflammatory agents to improve symptoms in patients with schizophrenia: an update. *Schizophr Bull,* 40**,** 181-91.

SPIES, C. M., BIJLSMA, J. W., BURMESTER, G. R. & BUTTGEREIT, F. 2010. Pharmacology of glucocorticoids in rheumatoid arthritis. *Curr Opin Pharmacol,* 10**,** 302-7.

SRIKANTH, S., RAVI, V., POORNIMA, K. S., SHETTY, K. T., GANGADHAR, B. N. & JANAKIRAMAIAH, N. 1994. Viral antibodies in recent onset, nonorganic psychoses: correspondence with symptomatic severity. *Biol Psychiatry,* 36**,** 517-21.

STAHN, C., LOWENBERG, M., HOMMES, D. W. & BUTTGEREIT, F. 2007. Molecular mechanisms of glucocorticoid action and selective glucocorticoid receptor agonists. *Mol Cell Endocrinol,* 275**,** 71-8.

STEFANSSON, H., OPHOFF, R. A., STEINBERG, S., ANDREASSEN, O. A., CICHON, S., RUJESCU, D., WERGE, T., PIETILAINEN, O. P., MORS, O., MORTENSEN, P. B., SIGURDSSON, E., GUSTAFSSON, O., NYEGAARD, M., TUULIO-HENRIKSSON, A., INGASON, A., HANSEN, T., SUVISAARI, J., LONNQVIST, J., PAUNIO, T., BORGLUM, A. D., HARTMANN, A., FINK-JENSEN, A., NORDENTOFT, M., HOUGAARD, D., NORGAARD-PEDERSEN, B., BOTTCHER, Y., OLESEN, J., BREUER, R., MOLLER, H. J., GIEGLING, I., RASMUSSEN, H. B., TIMM, S., MATTHEISEN, M., BITTER, I., RETHELYI, J. M., MAGNUSDOTTIR, B. B., SIGMUNDSSON, T., OLASON, P., MASSON, G., GULCHER, J. R., HARALDSSON, M., FOSSDAL, R., THORGEIRSSON, T. E., THORSTEINSDOTTIR, U., RUGGERI, M., TOSATO, S., FRANKE, B., STRENGMAN, E., KIEMENEY, L. A., GENETIC, R., OUTCOME IN, P., MELLE, I., DJUROVIC, S., ABRAMOVA, L., KALEDA, V., SANJUAN, J., DE FRUTOS, R., BRAMON, E., VASSOS, E., FRASER, G., ETTINGER, U., PICCHIONI, M., WALKER, N., TOULOPOULOU, T., NEED, A. C., GE, D., YOON, J. L., SHIANNA, K. V., FREIMER, N. B., CANTOR, R. M., MURRAY, R., KONG, A., GOLIMBET, V., CARRACEDO, A., ARANGO, C., COSTAS, J., JONSSON, E. G., TERENIUS, L., AGARTZ, I., PETURSSON, H., NOTHEN, M. M., RIETSCHEL, M., MATTHEWS, P. M., MUGLIA, P., PELTONEN, L., ST CLAIR, D., GOLDSTEIN, D. B., STEFANSSON, K. & COLLIER, D. A. 2009. Common variants conferring risk of schizophrenia. *Nature,* 460**,** 744-7.

STEINER, J., WALTER, M., GLANZ, W., SARNYAI, Z., BERNSTEIN, H. G., VIELHABER, S., KASTNER, A., SKALEJ, M., JORDAN, W., SCHILTZ, K., KLINGBEIL, C., WANDINGER, K. P., BOGERTS, B. & STOECKER, W. 2013. Increased prevalence of diverse N-methyl-D-aspartate glutamate receptor antibodies in patients with an initial diagnosis of schizophrenia: specific relevance of IgG NR1a antibodies for distinction from N-methyl-D-aspartate glutamate receptor encephalitis. *JAMA Psychiatry,* 70**,** 271-8.

TANDON, R., NASRALLAH, H. A. & KESHAVAN, M. S. 2010. Schizophrenia, "just the facts" 5. Treatment and prevention. Past, present, and future. *Schizophr Res,* 122**,** 1-23.

TORREY, E. F., BARTKO, J. J., LUN, Z. R. & YOLKEN, R. H. 2007. Antibodies to Toxoplasma gondii in patients with schizophrenia: a meta-analysis. *Schizophr Bull,* 33**,** 729-36.

TWEEDIE, D., SAMBAMURTI, K. & GREIG, N. H. 2007. TNF-alpha inhibition as a treatment strategy for neurodegenerative disorders: new drug candidates and targets. *Curr Alzheimer Res,* 4**,** 378-85.

TYRING, S., GOTTLIEB, A., PAPP, K., GORDON, K., LEONARDI, C., WANG, A., LALLA, D., WOOLLEY, M., JAHREIS, A., ZITNIK, R., CELLA, D. & KRISHNAN, R. 2006. Etanercept and clinical outcomes, fatigue, and depression in psoriasis: double-blind placebo-controlled randomised phase III trial. *Lancet,* 367**,** 29-35.

VAN BERCKEL, B. N., BOSSONG, M. G., BOELLAARD, R., KLOET, R., SCHUITEMAKER, A., CASPERS, E., LUURTSEMA, G., WINDHORST, A. D., CAHN, W., LAMMERTSMA, A. A. & KAHN, R. S. 2008. Microglia activation in recent-onset schizophrenia: a quantitative (R)-[11C]PK11195 positron emission tomography study. *Biol Psychiatry,* 64**,** 820-2.

VAN HAREN, N. E., HULSHOFF POL, H. E., SCHNACK, H. G., CAHN, W., MANDL, R. C., COLLINS, D. L., EVANS, A. C. & KAHN, R. S. 2007. Focal gray matter changes in schizophrenia across the course of the illness: a 5-year follow-up study. *Neuropsychopharmacology,* 32**,** 2057-66.

VAN STAA, T. P., LEUFKENS, H. G. & COOPER, C. 2002. The epidemiology of corticosteroid-induced osteoporosis: a meta-analysis. *Osteoporos Int,* 13**,** 777-87.

WEI, L., MACDONALD, T. M. & WALKER, B. R. 2004. Taking glucocorticoids by prescription is associated with subsequent cardiovascular disease. *Ann Intern Med,* 141**,** 764-70.

WEISER, M., BURSTEIN, S., FODOREANU, L., SHIRITA, R., TALAU, G., CIRJALIU, D., FUND, N., YOLKEN, R., DAVIS, J., DAVIDSON, M. 2014. Positive Symptoms respond to add-on aspirin in schizophrenia patients with high sera CRP levels: a post-hoc analysis of an RCT. . *Schizophr Res,* 153**,** S79.

WHO 2001. The world health report 2001 - Mental Health: New Understanding, New Hope.

WIUM-ANDERSEN, M. K., ORSTED, D. D. & NORDESTGAARD, B. G. 2014. Elevated C-reactive protein associated with late- and very-late-onset schizophrenia in the general population: a prospective study. *Schizophr Bull,* 40**,** 1117-27.

YATES, C. J., FOURLANOS, S., COLMAN, P. G. & COHNEY, S. J. 2014. Divided dosing reduces prednisolone-induced hyperglycaemia and glycaemic variability: a randomized trial after kidney transplantation. *Nephrol Dial Transplant,* 29**,** 698-705.

ZEPHIR, H., DE SEZE, J., DUJARDIN, K., DUBOIS, G., CABARET, M., BOUILLAGUET, S., FERRIBY, D., STOJKOVIC, T. & VERMERSCH, P. 2005. One-year cyclophosphamide treatment combined with methylprednisolone improves cognitive dysfunction in progressive forms of multiple sclerosis. *Mult Scler,* 11**,** 360-3.

ZEPHIR, H., DE SEZE, J., DUJARDIN, K., DUBOIS, G., CABARET, M., BOUILLAGUET, S., FERRIBY, D., STOJKOVIC, T. & VERMERSCH, P. 2008. [Cognitive impact of mitoxantrone and methylprednisolone in multiple sclerosis: an open label study]. *Rev Neurol (Paris),* 164**,** 47-52.

1. **Supplementary figures**

**Figure 6.** Fasting blood glucose (mmol/L) and HbA1c (mmol/L) for the prednisolone and placebo groups

**Figure I, a-f.** Leukocytes and differential count (10^9^/L)

**Figure II.** Sensitivity analysis: PANSS total and subscores in models adjusted for age, sex, and baseline

**Figure III.** Sensitivity analysis without one patient in the prednisolone group with autoimmune disease who exhibited high CRP

**Figure IV.** Sensitivity analysis without the outlier in the placebo group, exhibiting markedly high PANSS at week 6

**Figure V.** PANSS total and subscores without the patient who prematurely terminated the prednisolone add-on due to Covid-19 symptoms

1. **CRP**

The individual CRP values at baseline are listed in the table below. The value 0 is given when the value is below lower limit of quantification (below 0.19 mg/L).

CRP-values (mg/L) at baseline

|  | Prednisolone | Placebo |
| --- | --- | --- |
| 1 | 14 | 1 |
| 2 | 0 | 0 |
| 3 | 2.6 | 0 |
| 4 | 3 | 1.1 |
| 5 | 1 | 1 |
| 6 | 0.5 | 1.4 |

1. **Table A. Patient-rated side effects (UKU)**

|  |  | Week 1 | 2 weeks | 3 weeks | 4 weeks | 5 weeks | 6 weeks |
| --- | --- | --- | --- | --- | --- | --- | --- |
| UKU1 | Placebo | 9 (2·1) | 12 (2·1) | 10·6 (2·1) | 9·6 (2·1) | 6 (2·1) | 9 (2·1) |
|  | Prednisolone | 4·2 (1·9) | 3·7 (2) | 3·7 (2) | 2·7 (2) | 2·2 (1·9) | 2·2 (1·9) |
|  | Difference (SD)  [p-value] | 4·8 (2·9) [0·128] | 8·3 (2·9) [0·02] | 6·9 (2·9) [0·044] | 6·9 (2·9) [0·044] | 3·8 (2·9) [0·217] | 6·8 (2·9) [0·042] |
|  |  |  |  |  |  |  |  |
| UKU2 | Placebo | 2·4 (1·2) | 5·2 (1·2) | 3·8 (1·2) | 1·4 (1·2) | 1·4 (1·2) | 1·4 (1·2) |
|  | Prednisolone | 1·7 (1·1) | 1·7 (1·2) | 0·9 (1·2) | 0·9 (1·2) | 0·5 (1·1) | 0·3 (1·1) |
|  | Difference (SD)  [p-value] | 0·7 (1·7) [0·672] | 3·5 (1·7) [0·071] | 2·9 (1·7) [0·125] | 0·5 (1·7) [0·781] | 0·9 (1·7) [0·605] | 1·1 (1·7) [0·541] |
|  |  |  |  |  |  |  |  |
| UKU3 | Placebo | 7·8 (1·6) | 5·6 (1·6) | 5 (1·6) | 4·6 (1·6) | 2 (1·6) | 2·8 (1·6) |
|  | Prednisolone | 2 (1·5) | 1·8 (1·5) | 1·2 (1·5) | 1·2 (1·5) | 1·3 (1·5) | 1·7 (1·5) |
|  | Difference (SD)  [p-value] | 5·8 (2·2) [0·025] | 3·8 (2·2) [0·115] | 3·8 (2·2) [0·115] | 3·4 (2·2) [0·152] | 0·7 (2·2) [0·764] | 1·1 (2·2) [0·611] |

Note. The UKU Side Effect Rating Scale consists of items clustered into four subgroups: psychiatric, neurological, autonomic, and “other” symptoms. The “other” group was not relevant here, thus three groups are listed.

1. **Impact of the Covid-19 pandemic on enrolment**

The outbreak of the novel coronavirus (Covid-19), with a state of emergency in Norway from 11 March 2020, affected the study inclusion severely. The add-on of an anti-inflammatory agent like prednisolone to otherwise immune-competent individuals seemed to be unethical before more knowledge about the virus was available. In agreement with the Regional Committees for Medical and Health Research Ethics, and the Norwegian Medicines Agency, study inclusion was put on hold from 11 March 2020.

No events compromising patient safety due to the Covid-19 outbreak were registered for the enrolled study participants. Nonetheless, when we re-initiated inclusion from 30 October 2020, some precautions were added to the protocol to ensure safety for all new study participants: They had to remain/be admitted in a psychiatric ward for the 14 first days of receiving study medication, and extra precautions were taken to ensure that patients were free of symptoms indicating possible viral infections before study medication was administered. Unfortunately, recruiting new participants in the year after the Covid-19-induced break proved very difficult, hence the study was prematurely terminated.
